# Effects of Fiber Type and Size on the Heterogeneity of Oxygen Distribution in Exercising Skeletal Muscle

**DOI:** 10.1371/journal.pone.0044375

**Published:** 2012-09-18

**Authors:** Gang Liu, Feilim Mac Gabhann, Aleksander S. Popel

**Affiliations:** 1 Systems Biology Laboratory, Department of Biomedical Engineering, School of Medicine, Johns Hopkins University, Baltimore, Maryland, United States of America; 2 Institute for Computational Medicine, Department of Biomedical Engineering, Johns Hopkins University, Baltimore, Maryland, United States of America; University of Arizona, United States of America

## Abstract

The process of oxygen delivery from capillary to muscle fiber is essential for a tissue with variable oxygen demand, such as skeletal muscle. Oxygen distribution in exercising skeletal muscle is regulated by convective oxygen transport in the blood vessels, oxygen diffusion and consumption in the tissue. Spatial heterogeneities in oxygen supply, such as microvascular architecture and hemodynamic variables, had been observed experimentally and their marked effects on oxygen exchange had been confirmed using mathematical models. In this study, we investigate the effects of heterogeneities in oxygen demand on tissue oxygenation distribution using a multiscale oxygen transport model. Muscles are composed of different ratios of the various fiber types. Each fiber type has characteristic values of several parameters, including fiber size, oxygen consumption, myoglobin concentration, and oxygen diffusivity. Using experimentally measured parameters for different fiber types and applying them to the rat extensor digitorum longus muscle, we evaluated the effects of heterogeneous fiber size and fiber type properties on the oxygen distribution profile. Our simulation results suggest a marked increase in spatial heterogeneity of oxygen due to fiber size distribution in a mixed muscle. Our simulations also suggest that the combined effects of fiber type properties, except size, do not contribute significantly to the tissue oxygen spatial heterogeneity. However, the incorporation of the difference in oxygen consumption rates of different fiber types alone causes higher oxygen heterogeneity compared to control cases with uniform fiber properties. In contrast, incorporating variation in other fiber type-specific properties, such as myoglobin concentration, causes little change in spatial tissue oxygenation profiles.

## Introduction

Oxygen transport from capillaries to muscle fibers plays an essential role in the maintenance of physiological functions of skeletal muscle across a wide range of conditions and various forms of exercise. Oxygen transport is regulated by convection in blood vessels and by diffusion across the vessel walls and into the interstitial space and parenchymal cells. Hemoglobin transports oxygen in the blood, while myoglobin serves as an oxygen storage and facilitates its diffusion inside myocytes. At resting conditions, oxygen consumption is less than the available oxygen delivered by the microvasculature, with nearly half of the oxygen returned to the venous circulation; thus, muscle at rest is typically oversupplied. During exercise, the oxygen consumption rate in skeletal muscle can increase as much as 50 fold compared to resting conditions [1]. The increased oxygen demand is partially compensated by the increased blood flow in tissue microcirculation (10 to 25 fold) [2], and the increase of oxygen extraction by the tissue. The mismatch between oxygen demand and oxygen supply may lead to regional tissue hypoxia, and prolonged hypoxia can result in angiogenesis (capillary growth from pre-existing vasculature), an adaptive response that leads to a decrease of oxygen diffusion distances [3]. Angiogenesis may also result from the elevated shear stress often associated with functional hyperemia [4]. Insufficient oxygen supply is a major downstream pathological effector of chronic ischemic diseases such as coronary artery disease and peripheral artery disease [5]. In those ischemic diseases, the obstruction of upstream blood vessels limits blood flow and convective oxygen transport.

Theoretical aspects of oxygen transport processes have been extensively studied. The goal has been to describe the non-uniform oxygen distribution in skeletal muscle tissue. In the pioneering work by August Krogh [6], an oxygen transport model (also called the Krogh-Erlang model) was developed to describe a single capillary supplying oxygen to a cylindrical volume of surrounding tissue. It was the first theoretical model used to understand oxygen transport to tissue and was built on a number of idealized assumptions including: constant oxygen consumption rate; uniform oxygen diffusivity; and homogeneous capillary distribution. Since then, many alternative oxygen transport models have been developed (reviewed in [7]–[9]) with a number of additional physiological features neglected in the Krogh-Erlang model, including: variable oxygen consumption rate [10]; myoglobin-facilitated diffusion [11]; flow redistribution [12]; axial oxygen diffusion in the tissue [13]; intravascular oxygen transport resistance [14], and pre- and post-capillary transport [15], [16]. In addition, oxygen transport models have been extended to include complex microvascular network geometry with capillary tortuosity and anastomoses [17]–[19]. The Krogh-Erlang model is informative; however because of the complexity and heterogeneity of tissue microarchitecture, it would be difficult to establish a single Krogh-type parameter to describe the fiber types or the vessels adjacent to them. Instead, we construct a spatially detailed heterogeneous model based on the experimentally reported values of capillary∶fiber ratio for specific fibers and report the distribution of oxygen across the tissue being simulated.

Skeletal muscle is composed of an array of fibers with capillaries running primarily parallel to the fiber direction. Muscles in the body have different functions and different fiber compositions; fiber type composition within a muscle is closely correlated to the function of that muscle. Fibers are commonly categorized as type I (slow oxidative twitch) and type II (fast twitch) based on contractile properties and oxidative capacity [20]–[22]. Type I fibers are mainly involved in aerobic activities and endurance exercise; they contain ample amounts of mitochondria and utilize mostly oxidative phosphorylation, making them fatigue-resistant. In addition, they are myoglobin-rich with a red appearance. Type II fibers, appearing whiter than the ‘red’ Type I fibers due to lower myoglobin expression, can be classified into three subtypes, Type IIa, IIb and IId/x. Type IIa (or oxidative fast twitch) fibers generate ATP through the glycolytic cycle but also have a high mitochondrial count, allowing them to obtain ATP through oxidative metabolism. They have higher contraction velocities compared to Type I fibers but are not fatigue-resistant. Type IIb (or glycolytic fast twitch) fibers have far fewer mitochondria and thus depend on glycolytic metabolism to generate ATP. They are activated when short and powerful bursts of contraction are required. Type IId/x fibers have properties intermediate between Type IIa and Type IIb. Type I and Type IIa fibers have higher oxidative capacities and therefore consume more oxygen than Type IId/x and Type IIb. These muscle fibers are distributed throughout the skeletal muscle tissue in varying proportions, depending on the muscle group involved. In addition, there is a correlation between the fiber type and the number of adjacent blood vessels: Type IIb fibers generally have fewer adjacent blood capillaries compared to Type I and Type IIa. In addition to histological and metabolic differences, muscle fibers also differ in their size and their components, with variations in the proportions of aqueous cytosol and lipid-rich membranes and droplets, allowing varying oxygen diffusivity in different fiber types [9].

The objective of our study is to assess the impact of fiber type composition on the heterogeneity of oxygen distribution in tissue using a computational oxygen transport model. Our working hypothesis was that fiber-type dependent characteristics may balance with the number of nearby capillaries to locally ‘match’ oxygen supply and demand. We further hypothesized that this matching may be more relevant in exercise conditions. One goal of the study was to use computational modeling techniques as a hypothesis testing tool to understand the role of each parameter. The computational model is an extension of our previously developed oxygen model (*see* Methods). We began by building several tissue geometries that incorporate fiber type-specific properties: at the tissue level this includes the proportion of each type of fiber present; at the fiber level, each fiber type has a different characteristic fiber size, oxygen consumption rate (*M*_c_), oxygen diffusivity (*D_O2_*), myoglobin concentration (*C_Mb_*), and number of surrounding capillaries. We constructed a set of tissue geometries; each geometry has a different combination of parameters set to be uniform and parameters set to be fiber type-specific. This allows us to capture the different effects of each parameter on spatial heterogeneity in the muscle. For each geometry, the oxygen transport model is used to simulate the spatial profiles of oxygen under exercise conditions of varying intensities. Our simulation results suggest a marked increase of spatial heterogeneity of oxygen due to fiber size distribution in a mixed muscle. The difference in oxygen consumption rates of different fiber types also causes higher oxygen heterogeneity compared to control cases with uniform fiber properties. In contrast, incorporating variation in other fiber type-specific properties, such as myoglobin concentration, causes little change in spatial tissue oxygenation profiles. To the best of our knowledge, this is the first computational model used to examine the importance of fiber type and size in tissue oxygen transport.

## Methods

We adapted our multiscale computational model of oxygen transport [18], [23], [24] to study the effects of fiber type and fiber size on the oxygen distribution and oxygen gradients in working muscle. Two new features were introduced into the model. First, we incorporated six variables representing experimentally-measured physiological properties of specific fiber types in skeletal muscle. These variables are fiber type composition, fiber size, *D_O2_*, *M*_c_, *C_Mb_* and capillary distribution. Second, the modified model describes the oxygen transport process using coupled partial differential equations in three regions: inside the muscle fibers, in the interstitial space, and inside the vascular space. Previously, fibers and interstitial space had not been treated separately.

The model consists of three components: the muscle geometry module, the microvascular blood flow module, and the oxygen transport module. We will describe each module, with the focus on the new model features in this study.

### Skeletal Muscle Geometry Module

We constructed a set of skeletal muscle tissue geometries. These constructions were done computationally, using a similar algorithm to that applied in previous studies [23]–[28], with modifications described below. The muscle in our model is the rat extensor digitorum longus (EDL) that has been used extensively in experimental studies of activity-induced angiogenesis. To assess the effects of fiber type, fiber size and also fiber-type dependent microvascular structures on oxygen distribution profiles, we specifically introduced four types of skeletal muscle geometry. Each geometry (designated G1–G4) incorporates an experimentally-observed number (or percentage) of each fiber type. However they differ as follows: G1, uniform fiber size and uniform capillary distribution; G2, uniform fiber size and fiber-type-specific capillary distribution; G3, non-uniform fiber size and uniform capillary distribution; and G4, non-uniform fiber size and fiber-type-specific capillary distribution.

**Figure 1:**
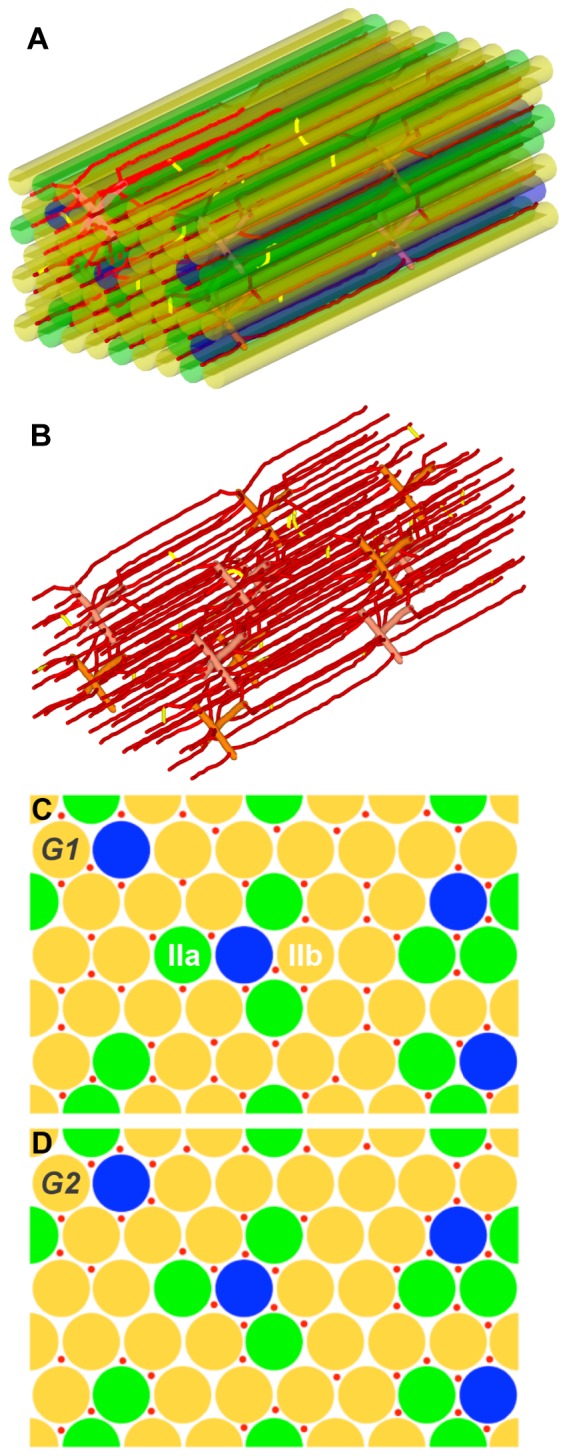
Muscle geometries with uniform-size fibers. **A**) An example of the 3D structure of skeletal muscle with uniform-size fibers and microvasculature. **B**) 3D structure of the microvasculature. **C**) 2D cross section of skeletal muscle with uniform-size fibers and uniform capillary distribution (G1). **D**) 2D cross section of skeletal muscle with uniform size fiber and fiber type dependent capillary distribution (G2). Fiber type I is shown in blue, type IIa in green and type IIb in yellow. Microvessels are shown in red.

**Figure 2:**
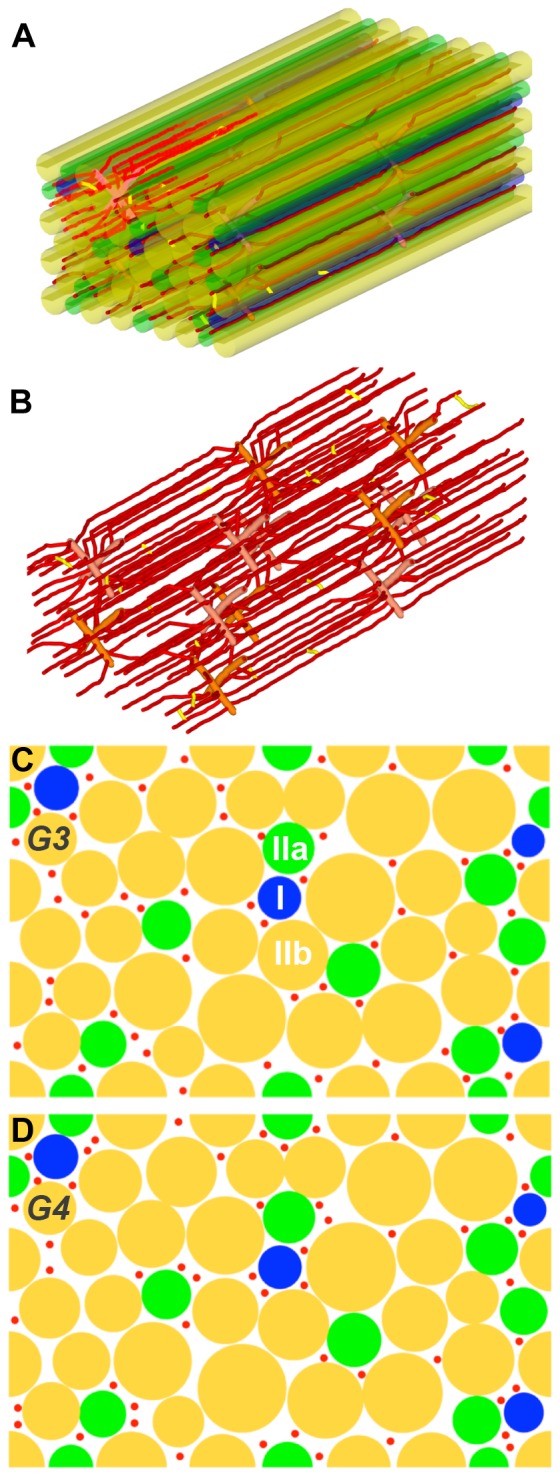
Muscle geometries with non-uniform-size fibers. **A**) An example of the 3D structure of skeletal muscle including non-uniform-size fibers and microvasculature. **B**) 3D structure of the microvasculature. **C**) 2D cross section of skeletal muscle with non-uniform size fibers and uniform capillary distribution (G3). **D**) 2D cross section of skeletal muscle with non-uniform size fibers and fiber-type-dependent capillary distribution (G4). Fiber type I is shown in blue, type IIa in green and type IIb in yellow. Microvessels are shown in red.

Computational generation of these 3D muscle geometries (Figures 1 and 2) is implemented as a two-step method, with fiber geometry constructed first followed by microvascular network insertion. The outline of the design algorithm is as follows: (1) In the rectangular cuboid tissue volume, muscle fibers are represented as right circular cylinders; (2) Microvessels, specifically capillaries, are placed between the muscle fibers; the vessels are divided into short segments and each segment is represented as a right circular cylinder; tortuosity and anastomoses are introduced to mimic physiological vascular networks by varying the orientation of adjoining segments; (3) Pre-capillary arterioles and post-capillary venules are placed in a staggered pattern to connect with the microvessel network; the capillaries originate from an arteriole and terminate at a venule approximately 350 µm away.

For fiber geometry with uniform-size fibers (G1 and G2), we place the fibers on a regular grid [23], [25]–[28]. To avoid edge effects, a number of fibers are bisected by the edges, but appear on the other side in a periodic fashion. The simulations apply periodic boundary conditions for oxygen diffusion. We then use a randomizing algorithm, combined with experimentally-observed rules, to assign fiber types to fibers. The fiber type composition is dependent on the muscle type, animal species, and individual differences. The proportion of fiber type I in the EDL ranges from 2 to 9%, fiber type IIa from 11 to 27%, and fiber type IIb from 61 to 82% [29]. For this model, we only consider fiber type I, fiber type IIa and fiber type IIb due to incomplete information about fiber type IId/x (for example, values for the myoglobin concentration and oxygen diffusion coefficient in this fiber type are not available). In our constructed geometries, we consider 48 fibers in the tissue volume (358×233×800 µm), of which 4 fibers are type I (8.3%), 11 are type IIa (22.9%) and 33 are type IIb (68.8%). Second, based on histochemical staining reported in the literature [29], there are two rules for fiber type distribution in rat EDL muscle: (1) type I fibers are not typically found next to each other; (2) type IIa fibers are typically found close to type I fibers.

For our tissue geometries with fiber-type-specific fiber sizes (G3 and G4), we use a hybrid simulated-annealing algorithm [30] to construct the fiber geometry. The optimization function is designed to find the best fit of several experimentally-observed physiological parameters. These parameters are: interstitial fluid volume fraction 6%–20% [31]–[33]; fiber type proportion 2–9%, 11–27%, 61–82% for fiber type I, IIa and IIb respectively [29]); the radii for fiber type I, type IIa, type IIb are 13, 16, and 23 µm respectively, based on reported fiber areas [34].

For the microvascular network in the tissue, capillary placement is also governed by a stochastic algorithm, augmented by several rules including: (1) the average capillary-to-fiber ratio (C∶F) is 1.1 (i.e., the total number of capillaries is about 52). The capillary density is 623/mm^2^, which is close to 650 (recalibrated value, number per fiber area including interstitial space, reported in [35]); (2) the radii of capillary, venule and artery segments are set as uniform in the vascular network (3, 6, 6 µm); and (3) the number of capillaries around a fiber (NCAF) is either specific (G2, G4) or non-specific (G1, G3) to fiber type, depending on the constructed geometry (i.e. uniform or heterogeneous fiber-type-specific microvascular distribution. For the uniform vascular network geometries (G1, G3), the average NCAF values for three fiber type groups are the same (approximately 3.24) and we also seek to minimize the variability of NCAF between individual fibers in the geometry (Fig. 1 and 2). For fiber-type-specific vascular geometries (G2, G4), we follow experimental observations in rat skeletal muscle that muscle fibers with a high oxidative potential are generally associated with a denser capillary network [36], [37], although not all studies agree with this finding [38]. In this type of fiber-type-specific geometry (Fig. 1 and 2), fiber type I has the largest number of surrounding capillaries, followed by type IIa and then type IIb. NCAF values for each fiber varies from 1 to 6. In addition, we also designed the geometry with a significant degree of heterogeneity in each fiber type group.

### Flow Module

We apply an in vivo hemorheological model [39] to calculate the blood flow rate (*Q*) and discharge hematocrit (*H_D_*) in each of the blood vessel segments in the muscle, which incude terminal arterioles, capillaries and collecting venules. The vascular network is digitized as a set of nodes (vascular bifurcations) and a set of vessel segments linking those nodes together. The governing equations for volumetric blood flow rate and red blood cell flow rate at the j^th^ node are:
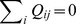


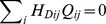
where *Q_ij_* and *H_D_*_ij_ are the volumetric flow rate and discharge hematocrit in vessel segment *ij*, a cylindrical segment whose ends are the *i*th and *j*th nodes of the network. Using the governing equations combined with empirical equations describing red blood cell-plasma separation at vascular bifurcations [39], we obtained a set of nonlinear algebraic equations for all *N* segments and the equations were solved for pressure and hematocrit, from which flow in each segment is calculated.

### Oxygen Module

We use a modified version of our previously published oxygen transport model to compute oxygen distribution in the tissue. In previous studies we assumed that muscle fibers and the interstitial space were a single tissue phase [18], [23]. Here the model consists of three partial differential equations, governing: intravascular oxygen transport; oxygen diffusion in interstitial space; and oxygen diffusion and consumption inside fibers.

Local oxygen tension in the fibers, *P_f_*, is governed by free oxygen diffusion, myoglobin-facilitated diffusion, and oxygen consumption by myofibers:
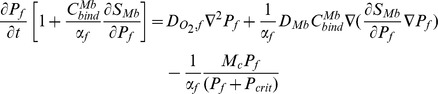
where *D_O2,f_* and *D_Mb_* are the diffusivities of oxygen (O_2_) and myoglobin (Mb) inside the fiber; *S_Mb_* is the oxygen-myoglobin saturation; *α_f_* is the oxygen solubility in the fiber; 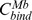 is the binding capacity of myoglobin for oxygen; *M*_c_ is the Michaelis-Menten-type maximal oxygen consumption rate; *P_crit_* is the critical *P_f_* at which oxygen consumption rate equals 50% of *M_c_*. *S_Mb_* is defined as *P_f_/*(*P_f_*+*P*_50,*Mb*_), assuming local binding equilibrium between oxygen and myoglobin, where *P_50,Mb_* is the *P_f_* corresponding to 50% myoglobin saturation with oxygen. It should be noted that the myoglobin-facilitated diffusion formulation is simplified in that it does not consider diffusion of myoglobin as a separate molecular species and assumes that myoglobin and oxygen are locally in chemical equilibrium; however, this assumption is justified *a posteriori* by the small effect of myoglobin under steady-state conditions.

Local oxygen tension in the interstitial space, *P_i_*, is governed only by free oxygen diffusion (we assume negligible consumption in this space):
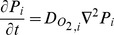
Oxygen transport within the blood vessels, *P_b_*, is governed by hemoglobin binding and blood convection:
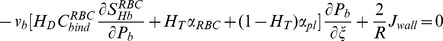
Here 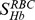 is the oxygen-hemoglobin saturation in blood vessel; *α_RBC_* and *α_pl_* are oxygen solubility in red blood cell and plasma, respectively; *P_b_* is the oxygen tension in blood plasma; *ν_b_* is the mean blood velocity (*ν_b_* = *Q*/(π*R* ^2^)); *H_T_* is the vessel (tube) hematocrit calculated from blood flow model; 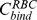 is binding capacity of hemoglobin with oxygen; *ξ* is the distance along a vessel's longitudinal axis; *J_wall_* is the capillary wall flux. 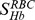 is defined as 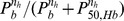 assuming binding equilibrium between oxygen and hemoglobin, where *P_50,Hb_* is the *P_b_* at 50% hemoglobin oxygen saturation.

Continuity of oxygen flux at the interface between blood vessels and interstitial space, and between muscle fiber and interstitial space, yields:
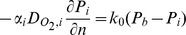


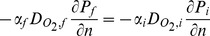
where *n* is the unit normal vector, and *k*_0_ is the mass transfer coefficient. This system of nonlinear partial differential equations was solved using the finite difference method, with a grid size of 1 micron as described in [23].

### Model parameters and simulation platform

Parameters used in the model are listed in Table 1 (fiber-type specific parameters) and Table 2 (non-fiber-type-specific parameters). Most of the model parameters were taken from experimental data for rat EDL; some were based on measurements in other muscle types; theoretical estimates were used when parameters are unavailable from literature. We use the multiscale modeling platform developed previously [24] to combine the three modules described above into an integrated model. This platform allows integration of different modules written in different programming languages and using different modeling methodologies. While the flow module remained unchanged from the previous version, the geometry and oxygen transport modules were updated based on the changes described above. The simulation experiments were run on an IBM cluster and each simulation was run on one node with 8 cores and 32 Gbyte memory. The oxygen transport module was implemented with OPENMP support and can be parallelized. JDK (Java Development Kit) 1.6.16 (Oracle, Redwood Shores, CA) is used as Java compiler, and Intel Fortran/C++ compiler suite 11.1 (Intel, Santa Clara, CA) is used as Fortran/C++ compiler.

**Table 1:** Fiber-type-specific parameters of the model.

Fiber type	% in EDL	*R_F_*	*C_Mb_*×10^4^	*M*_c_/*M_c,IIb_* *	*D_O2_*×10^5^
**Type I**	8.8[^29^]	13.6[^34^]	41[^51^]	2.08[^48^]	3.41[^52^]
**Type IIa**	22.9[^29^]	16.2[^34^]	20[^51^]	1.76[^48^]	1.96[^52^]
**Type IIb**	68.8[^29^]	22.8[^34^]	6.24[^51^]	1[^48^]	1.15[^52^]

Units: fiber radius (*R_F_*), µm; myoglobin concentration (*C_Mb_*), ml O_2_ ml^−1^; oxygen consumption rate (*M*_c_), ml O_2_ ml^−1^ s^−1^; oxygen diffusivity (*D_O2_*), cm^2^ s^−1^.

* : normalized to Type IIb.

**Table 2:** Non-fiber-type-specific parameters of the model.

Parameter	Description	Value	Unit	Reference
*P_50,Mb_*	PO*_2_* for 50% myoglobin O_2_ saturation	5.3	mmHg	[53]
*P*_50,Hb_	PO*_2_* for 50% hemoglobin O_2_saturation	37	mmHg	[54]
*n_h_*	hemoglobin O_2_ saturation Hill coefficients	2.7	-	[54]
*S_O2A_*	O_2_ saturation for arteriolar inlets	0.68	*-*	[23]
*D_Mb_*	Myoglobin diffusivity in tissue	3×10^−7^	cm^2^ s^−1^	[55]
*D_O2,i_* #	*D*_O2_ in interstitium	2.4×10^−5^	cm^2^ s^−1^	[56]
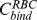	Hemoglobin O_2_-binding capacity	0.52	ml O_2_ (dl RBC)^−1^	[57]
*α_RBC_*	O_2_ solubility inside RBC	3.38×10^−5^	ml O_2_ ml^−1^ mmHg^−1^	[58]
*α_pl_*	O_2_ solubility in plasma	2.82×10^−5^	ml O_2_ ml^−1^ mmHg^−1^	[59]
*α_i_* #	O_2_ solubility in interstitium	2.80×10^−5^	ml O2 ml^−1^ mmHg^−1^	[56]
*α_f_*	O_2_ solubility in myocyte	3.89×10^−5^	ml O2 ml^−1^ mmHg^−1^	[56], [60]
*P_in_*	Inlet pressure	10	mmHg	[23]
*H_d,in_*	Inlet hematocrit	0.4	-	[23]

# : new model parameters in our study, other parameters (without superscript #) are the same as used in previous studies [17], [23].

## Results

We computationally evaluated the contribution of fiber type composition and fiber type-specific parameters to spatial heterogeneity of tissue oxygenation. These parameters include: fiber type-dependent oxygen consumption (*M*_c_), oxygen diffusivity in fiber (*D_O2_*), myoglobin concentration (*C_Mb_*), fiber size, and number of capillaries around a fiber (NCAF). To understand the effects of each factor separately as well as their combined effects, we ran a series of computational experiments to examine the heterogeneity of oxygen distribution in skeletal muscle during exercise in different muscle geometries (G1–G4) and with scenarios varying a number of the other factors under consideration (S1–S5). The annotations for each simulation are summarized in Table 3. For example, G3S1 refers to G3 geometry (non-uniform fiber size and uniform capillary distribution) with scenario S1 (uniform *M_c_*, *D_O2_*, and *C_Mb_*). The corresponding microvascular and fiber structures are shown in Figs. 1C (G1), 1D (G2), 2C (G3) and 2D (G4), representing the same location in networks (z = 400 µm). The three-dimensional geometry of G1 is shown in Fig. 1A,B and that of G3 in Fig. 2A,B. The number of capillaries around a fiber (NCAF) for each of the simulation geometries is shown in Table S1. In the following sections, we report the mean and the coefficient of variation (CV) of the tissue oxygen profile as a measure of the heterogeneity in the tissue for each simulation.

**Table 3:** List of simulation annotations.

Tissue Geometry & Scenario	Uniform Fiber Size	Uniform NCAF	Uniform *M*_c_	Uniform *D_O2_*	Uniform *C_Mb_*
**G1S1**	Yes	Yes	Yes	Yes	Yes
**G1S2**	Yes	Yes	No	No	No
**G2S1**	Yes	No	Yes	Yes	Yes
**G2S2**	Yes	No	No	No	No
**G3S1**	No	Yes	Yes	Yes	Yes
**G3S2**	No	Yes	No	No	No
**G3S3**	No	Yes	No	Yes	Yes
**G3S4**	No	Yes	Yes	No	Yes
**G3S5**	No	Yes	Yes	Yes	No
**G4S1**	No	No	Yes	Yes	Yes
**G4S2**	No	No	No	No	No
**G4S3**	No	No	No	Yes	Yes
**G4S4**	No	No	Yes	No	Yes
**G4S5**	No	No	Yes	Yes	No

It is worth noting that microvascular hemodynamics is a well-known important determinant of tissue oxygenation and it is not the focus of this study. Our blood flow simulations suggest that these vascular networks in two geometries (G1 and G2) share similar distribution patterns of blood flow velocity and hematocrit, and have similar total blood volume flow rates (see Table S2 and Fig. S1). In addition, vascular networks G3 and G4 share similar microvascular blood flow and red blood cell distribution (Table S2 and Fig. S2).

### Capillary distribution is an important determinant of oxygen distribution

**Figure 3:**
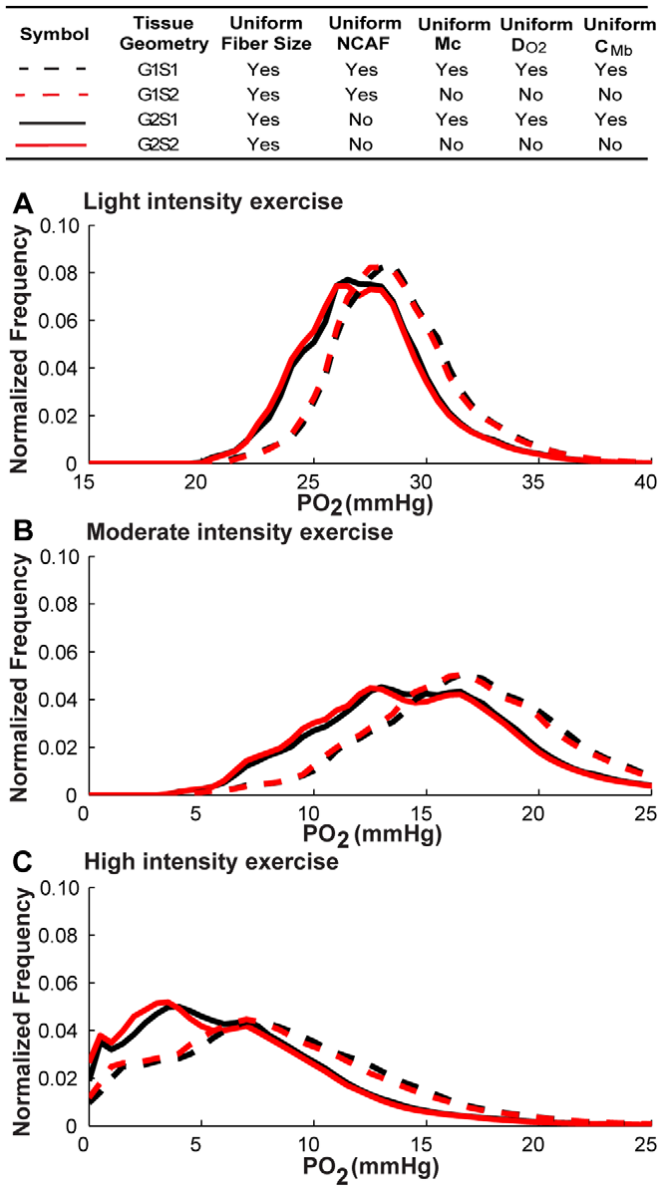
PO_2_ histograms for muscle geometries with uniform-size fibers under exercising conditions. Fiber PO_2_ probability distribution profiles in muscle tissue under **A**) light intensity exercise conditions, volume-averaged *M*_c_ = 3.34×10^−4^ mlO_2_ ml^−1^ s^−1^; **B**) moderate intensity exercise conditions, volume-averaged *M_c_* = 6.68×10^−4^ mlO_2_ ml^−1^ s^−1^ ; and **C**) high intensity exercise conditions, volume-averaged *M*_c_ = 1.02×10^−3^ mlO_2_ ml^−1^ s^−1^, for simulation cases G1S1, G1S2, G2S1, G2S2. Dotted lines are simulation results for G1 geometry, while solid lines are results for G2 geometry. Black lines are the simulation scenarios S1 with uniform fiber-type properties (*M*_c_, *D_O2_*, *C_Mb_*) and red lines are the simulation scenarios (S2 using fiber-type-specific parameters (except size) for different fiber types.

For the G1 and G2 geometries (uniform fiber size; Fig. 1) we first computed the steady-state tissue oxygen distribution without consideration for fiber type composition, not varying O_2_ consumption rates, O_2_ diffusivities, and myoglobin concentrations in different fiber types. In other words, uniform fibers with the same parameters: volume-averaged *M_c_* (3.34, 6.68, 10.02×10^−4^ mlO_2_ ml^−1^ s^−1^ for low, moderate, high intensity exercise), *D_O2_* (1.72×10^−5^ mlO_2_ ml^−1^ s^−1^), and *C_Mb_* (5.7×10^−3^ mlO_2_ ml^−1^ s^−1^) were used for every fiber in the simulation. In this case, with light intensity exercise, an increase in the heterogeneity of microvascular structure (G2 compared to G1), and therefore in the heterogeneity of oxygen supply, is predicted to lead to a slight increase in the heterogeneity of O_2_ (CV_G1S1_: 0.09, CV_G2S1_: 0.10) and a slight lowering of mean tissue PO_2_ level (P_G1S1_: 28.9 mmHg, P_G2S1_: 27.6 mmHg) (Fig. 3 and Table 4). In exercise of moderate or high intensity, the increase in oxygen consumption levels causes even greater heterogeneity. When capillaries are non-uniformly distributed around the fibers (Fig. 3, solid lines), the variation further increases; mean PO_2_ levels decrease from 17.4 to 15.1 mmHg (moderate intensity exercise), and from 8.84 to 6.82 mmHg (high intensity exercise). These results indicate that capillary distribution in muscle tissue affects non-uniform oxygen distribution and heterogeneity.

**Table 4:** Effects of fiber type properties on oxygen (PO_2_) distribution.

Tissue Geometry*	Exercise**	*P_mean_*	*P_min_*	*P_max_*	*CV(P)*	*%P<1* ***	*%P<2*
G1S1	low	28.94	21.21	42.91	0.09	0.00	0.00
G1S1	medium	17.40	4.48	40.13	0.26	0.00	0.00
G1S1	high	8.84	0.06	37.64	0.56	0.02	0.07
G1S2	low	28.85	21.39	42.93	0.09	0.00	0.00
G1S2	medium	17.18	4.55	40.25	0.26	0.00	0.00
G1S2	high	8.50	0.04	37.82	0.59	0.03	0.08
G2S1	low	27.62	20.23	42.51	0.10	0.00	0.00
G2S1	medium	15.10	3.69	39.27	0.30	0.00	0.00
G2S1	high	6.82	0.08	36.46	0.65	0.05	0.12
G2S2	low	27.50	20.37	42.49	0.10	0.00	0.00
G2S2	medium	14.84	3.65	39.38	0.31	0.00	0.00
G2S2	high	6.56	0.06	36.63	0.68	0.06	0.14

* see Table 3 for geometry and scenario annotations.

** Volume-averaged oxygen consumption (*M*_c_) in exercise: low, 3.34×10^−4^ ml O_2_ ml^−1^; medium, 6.68×10^−4^ ml O_2_ ml^−1^; high, 10.02×10^−4^ ml O_2_ ml^−1^.

*** % of tissue that is hypoxic, i.e. with PO_2_<1 or 2 mmHg.

### Fiber type composition does not contribute significantly to oxygen distribution

We then computed steady-state tissue oxygen distribution in geometries G1 and G2 when fiber type composition and varying *M*_c_, *D_O2_*, and C*_Mb_* in different fiber types (fiber type-specific parameters shown in Table 1) are considered; in other words, all fiber-type-specific parameters except for size. The simulation results with fiber type composition considered (scenario S2) were compared to uniform properties (scenario S1) (Table 4). Fig. 3 shows the histogram of muscle fiber PO_2_ in two muscle geometries (G1 and G2). Table 4 summarizes the key characteristics from all the simulation results of PO_2_ distribution under different intensities of exercise in G1 and G2.

At all levels of exercise, the difference in oxygen distribution between the heterogeneous fiber-type specific parameters (S2) and the uniform fiber parameters (S1, control cases) is minimal (Fig. 3, red vs. black lines). This includes the mean, range and variance of oxygen levels and the portion of tissue that is hypoxic, i.e. with PO_2_<1 or 2% (Table 4). For example, compared to the control cases with uniform fiber properties (i.e. G1S1, G2S1), the effect of fiber type composition on oxygen spatial heterogeneity and mean PO_2_ level is small when muscles are stimulated with light or moderate exercise intensity (e.g., for light exercise, CV for the geometry with uniform capillary distribution, CV_G1S2/S1_: ∼0.09, CV for the geometry with fiber-type-specific capillary distribution, CV_G2S2/S1_: ∼0.10, P_G1S2/S1_: ∼28.9 mmHg, P_G2S2/S1_: ∼27.6 mmHg). Under high intensity exercise conditions, oxygen spatial heterogeneity changes slightly compared to control cases (i.e. G1S1, G2S1) (CV_G1S2/S1_: 0.56 vs. 0.59; CV_G2S2/S1_ : 0.65 vs. 0.68) and mean PO_2_ remains at the same levels (PO_2 G1S2/S1_: 8.8 vs. 8.5 mmHg, high;PO_2 G2S2/S1_: 6.8 vs. 6.6 mmHg, high). The oxygen distribution is much more dependent on capillary distribution (Fig. 3, solid vs. dotted lines) than on heterogeneity of fiber properties (Fig. 3, red vs. black lines).

### Non-uniform fiber size significantly enhances the heterogeneity in oxygen distribution

**Figure 4:**
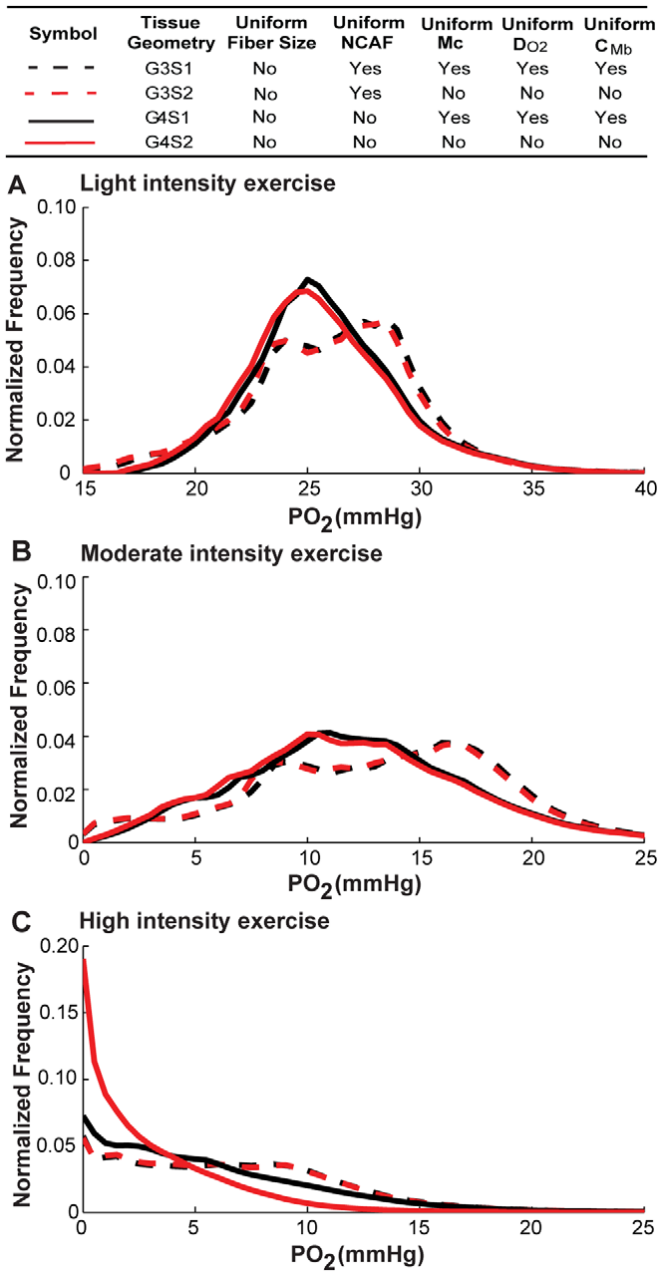
PO_2_ histograms for muscle geometries with non-uniform-size fibers under exercise conditions. PO_2_ probability distribution profiles for all points within fibers in muscle tissues under **A**) light intensity exercise. **B**) moderate intensity exercise. **C**) high intensity exercise, for simulation cases G3S1, G3S2, G4S1, G4S2. Dotted lines are simulation results for G3 geometry, while solid lines are results for G4 geometry. Black lines are the simulation scenarios S1 and red lines are the simulation scenarios S2.

**Figure 5:**
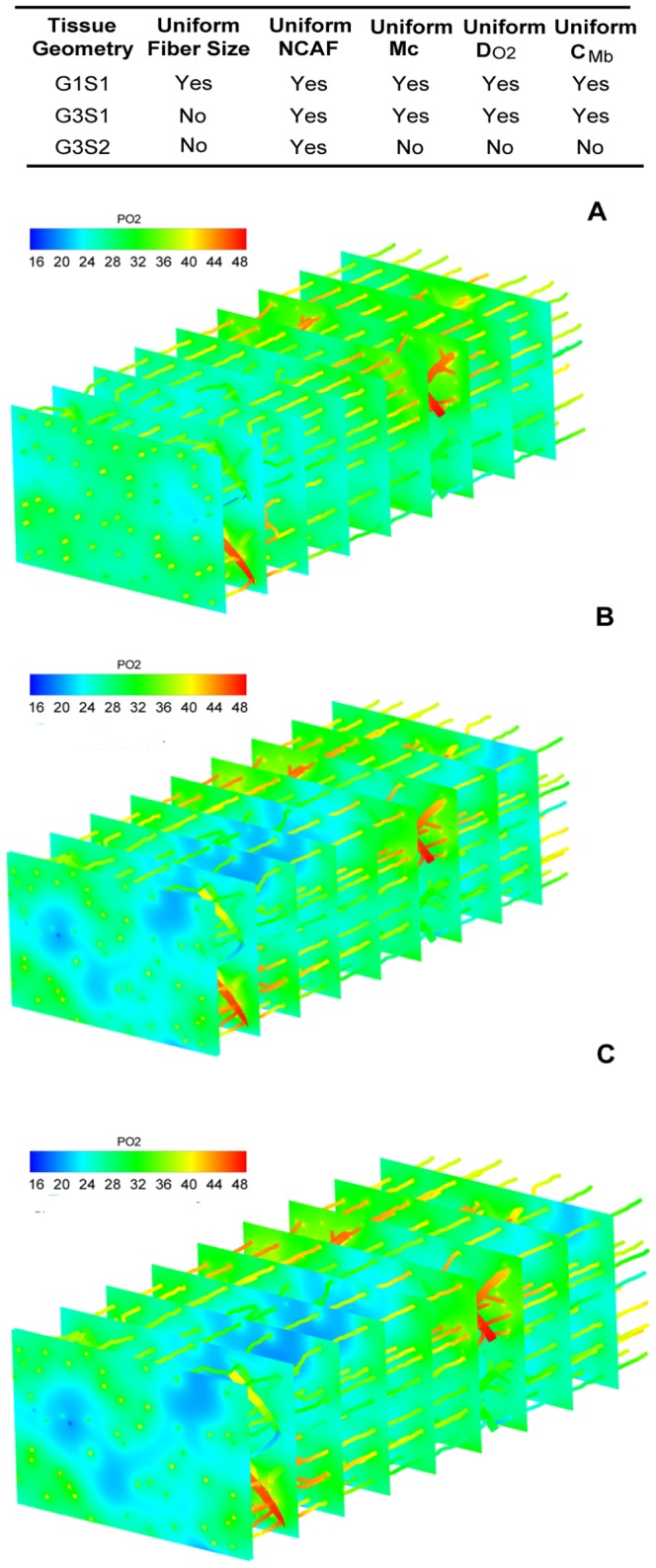
3D PO_2_ distribution in skeletal muscle. 3D tissue PO_2_ profiles in muscle tissues at light intensity exercise conditions for geometries **A**) G1S1; **B**) G3S1; **C**) G3S2.

To investigate the effect of fiber size on oxygen distribution in the EDL, we constructed geometries G3 and G4 (Fig. 2C,D), with non-uniform size fibers within the same dimension cuboid (358×233×800 µm^3^) and the same total fiber volume (79%) as G1 and G2 (Fig. 1C,1D). We first compared the tissue PO_2_ profile in geometry G3S1 with heterogeneous fiber sizes to the profile in geometry G1S1 with homogeneous fiber sizes. Both of them use uniform fiber type properties (scenario S1, i.e., *M*_c_, *D_O2_*, *C_Mb_*) and have uniform capillary distribution. Our oxygen transport simulation results suggest that fiber size distribution in muscle geometry plays an important role in determining tissue oxygen profile and spatial heterogeneity. Figure 4 shows that PO_2_ in heterogeneous fiber size geometry is much more broadly distributed compared to the control case (i.e., G1S1) with uniform size fiber under all exercise conditions (G3S1 vs. G1S1, black dashed lines in Fig. 4 vs. Fig. 3; 3D graphical representation shown in Fig. 5A,B). Heterogeneous fiber size distribution shifts tissue PO_2_ to lower values (mean PO_2_ from 28.9 to 26.5 mmHg, light exercise; 17.1 vs. 13.6 mmHg, moderate; 8.84 vs. 6.96 mmHg, high), and increases its spatial heterogeneity (0. 09 vs. 0.14, light; 0.26 vs. 0.42, moderate; 0.56 vs. 0.71, high). Under high intensity exercise conditions, the proportion of hypoxic tissue is much larger than in control case (18% vs. 7%).

We further examined the effects of fiber-type-specific properties on oxygen distribution in the non-uniform size fiber geometries (geometries G3 and G4, results in Figure 4 and Table 5). Conclusions similar to those described above can be drawn from these data. First, oxygen distribution is sensitive to local capillary distribution around the fibers. However, their effects are different from previous case with uniform size fiber distribution (Fig. 4, solid vs. dashed lines). With fiber-type-dependent capillary distribution (smaller fibers have more adjacent capillaries), the coefficients of variation of the tissue PO_2_ profiles under light exercise conditions are slightly decreased compared to the tissue with uniform capillary distribution (CV: 0.14 vs. 0.12, light; 0.42 vs. 0.42, moderate), and their mean PO_2_ levels shift to slightly lower values (PO_2_: 26.47 vs. 26.09 mmHg, light; 13.56 vs. 12.54 mmHg). Second, with the consideration of all fiber type properties (scenario S2) and fiber size, tissue PO_2_ profiles do not change significantly from tissue PO_2_ profiles computed using the uniform fiber properties. This conclusion is based on the comparisons of tissues PO_2_ in geometries with uniform/non-uniform capillary distribution in Fig. 4 and three-dimensional graphical representation in Fig. 5B,C. There are only slight differences between black and red lines (including dashed and solid cases) at all exercise intensities, with one exception: under high intensity exercise and with fiber-type-specific capillary distribution, heterogeneous fiber type composition increases spatial heterogeneity (CV: 0.81 vs. 1.00) and reduces its tissue PO_2_ level (PO_2_: 5.93 vs. 3.22 mmHg).

**Table 5:** Effects of fiber type properties and fiber size on oxygen (PO_2_) distribution.

Tissue Geometry*	Exercise**	*P_mean_*	*P_min_*	*P_max_*	*CV(P)*	*%P<1* ***	*%P<2*
G3S1	low	26.47	15.20	44.05	0.14	0.00	0.00
G3S1	medium	13.56	0.10	41.64	0.42	0.01	0.03
G3S1	high	6.96	0.00	38.84	0.71	0.10	0.18
G3S2	low	26.22	14.64	43.99	0.14	0.00	0.00
G3S2	medium	13.34	0.10	41.51	0.43	0.01	0.03
G3S2	high	6.87	0.00	38.65	0.71	0.10	0.18
G4S1	low	26.09	17.48	43.59	0.12	0.00	0.00
G4S1	medium	12.54	0.53	40.77	0.42	0.00	0.01
G4S1	high	5.93	0.01	38.32	0.81	0.13	0.23
G4S2	low	25.81	16.91	43.63	0.13	0.00	0.00
G4S2	medium	12.27	0.50	40.88	0.43	0.00	0.01
G4S2	high	3.22	0.00	32.43	1.00	0.30	0.47

* see Table 3 for geometry and scenario annotations.

** Volume-averaged oxygen consumption (*M*c) in exercise: low, 3.34×10^−4^ ml O_2_ ml^−1^; medium, 6.68×10^−4^ ml O_2_ ml^−1^; high, 10.02×10^−4^ ml O_2_ ml^−1^.

*** % of tissue that is hypoxic, i.e. with PO_2_<1 or 2 mmHg.

**Figure 6:**
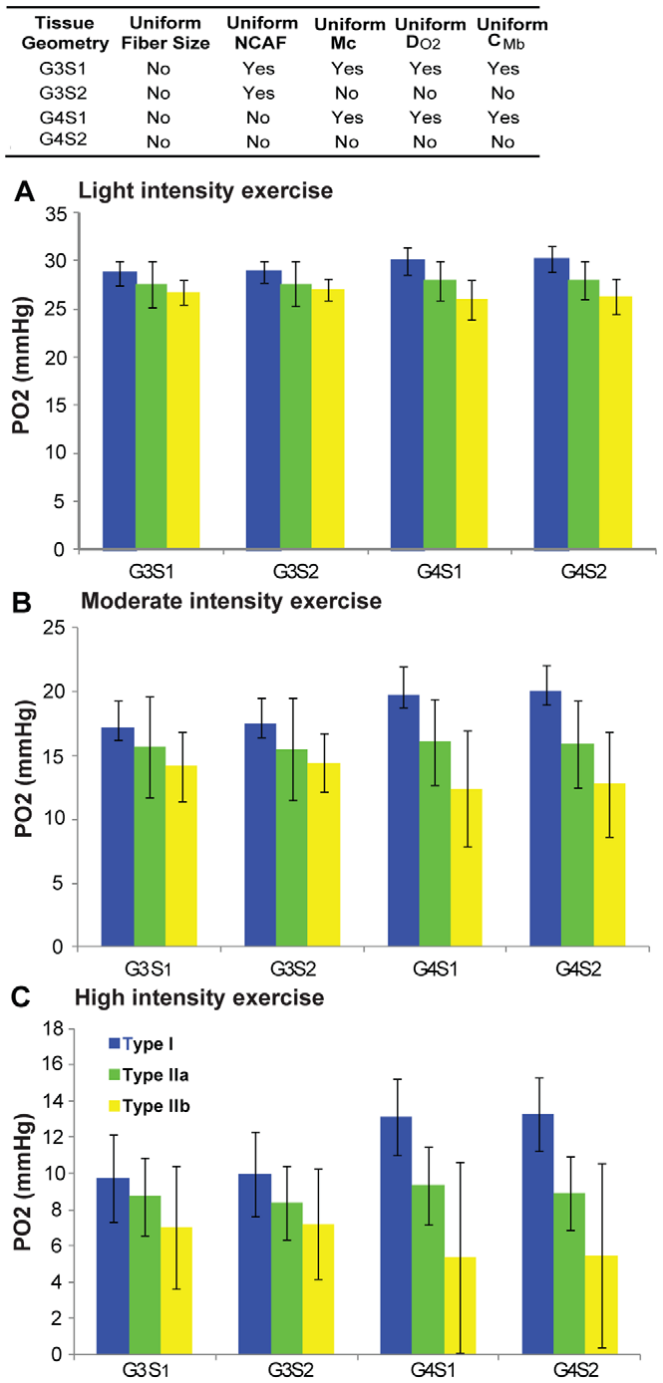
Distribution of average PO_2_ levels of individual fibers in skeletal muscle under exercise conditions. Average (across all fibers of each type) of the mean PO_2_ levels of individual fibers under **A**) light intensity exercise; **B**) moderate intensity exercise; and **C**) high intensity exercise, for simulation cases G3S1, G3S2, G4S1 and G4S2. Type I is shown in blue, type IIa in green, type IIb in yellow.

We further studied the distribution of average PO_2_ for each fiber from three fiber type groups under various cases (Fig. 6). This shows that under light intensity exercise, the difference in average fiber PO_2_ level among the three fiber type groups is low. With the increase of exercise intensity (to moderate and high intensity), fibers of type I have higher local oxygenation levels than types IIa and IIb. With fiber-type specific capillary distribution (G4 vs. G3), the oxygenation levels increase for oxidative fibers type I and type IIa, but decrease for type IIb. Therefore, at high intensity exercise conditions, the difference of PO_2_ levels between fiber type IIb and fiber type I increased significantly, suggesting that local oxygen supply matches local demand well for oxidative fiber but not glycolytic fiber in exercise. Greater heterogeneity of average PO_2_ level in each fiber type was also observed for higher exercise intensities and with capillary heterogeneity.

### Fiber-type-specific *M*_c_ and *D_O2_*, but not *C_Mb_*, cause greater heterogeneity in oxygen distribution

**Figure 7:**
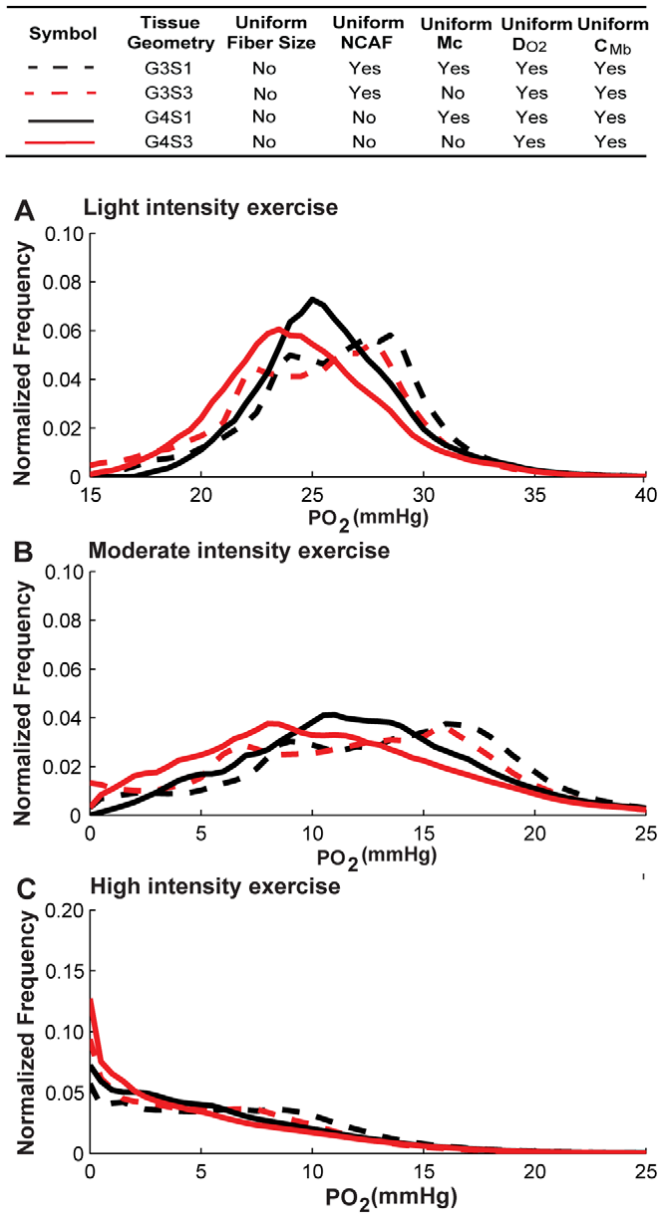
Effects of variation of oxygen consumption rate in different fiber types on PO_2_ histograms. Fiber PO_2_ probability distribution profiles in muscle tissues under **A**) light intensity exercise; **B**) moderate intensity exercise; and **C**) high intensity exercise, for simulation cases G3S1, G3S3, G4S1 and G4S3. Dotted lines are simulation results from G3 geometry, solid lines are results from G4 geometry. Black lines are the simulation scenarios S1 and red lines are the simulation scenarios (S3) when *M*_c_ is fiber-type-dependent while others use uniform values for different fiber types.

**Figure 8:**
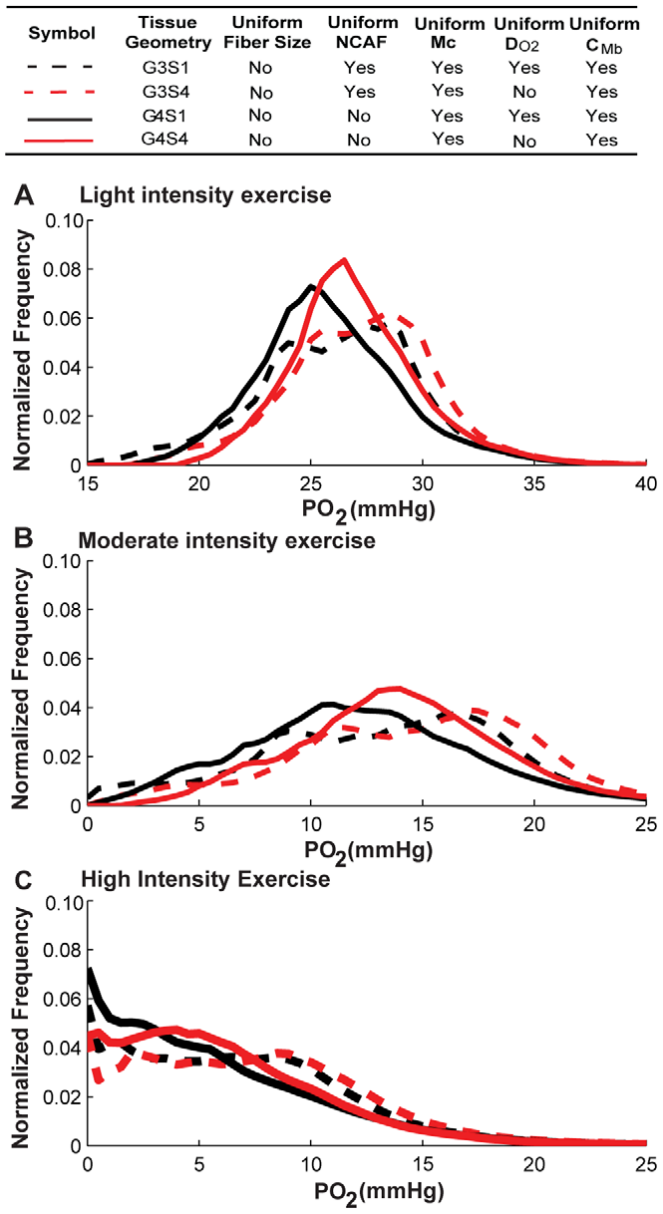
Effects of variation of oxygen diffusivity in different fiber types on PO_2_ histograms. Fiber PO_2_ probability distribution profiles in muscle tissues under **A**) light intensity exercise; **B**) moderate intensity exercise; and **C**) high intensity exercise, for simulation cases G3S1, G3S4, G4S1 and G4S4. Dotted lines are simulation results for G3 geometry, solid lines are results for G4 geometry. Black lines are the simulation scenarios S1 and red lines are simulations scenarios S4 when *D_O2_* is fiber-type-dependent while others use uniform values for different fiber types.

**Figure 9:**
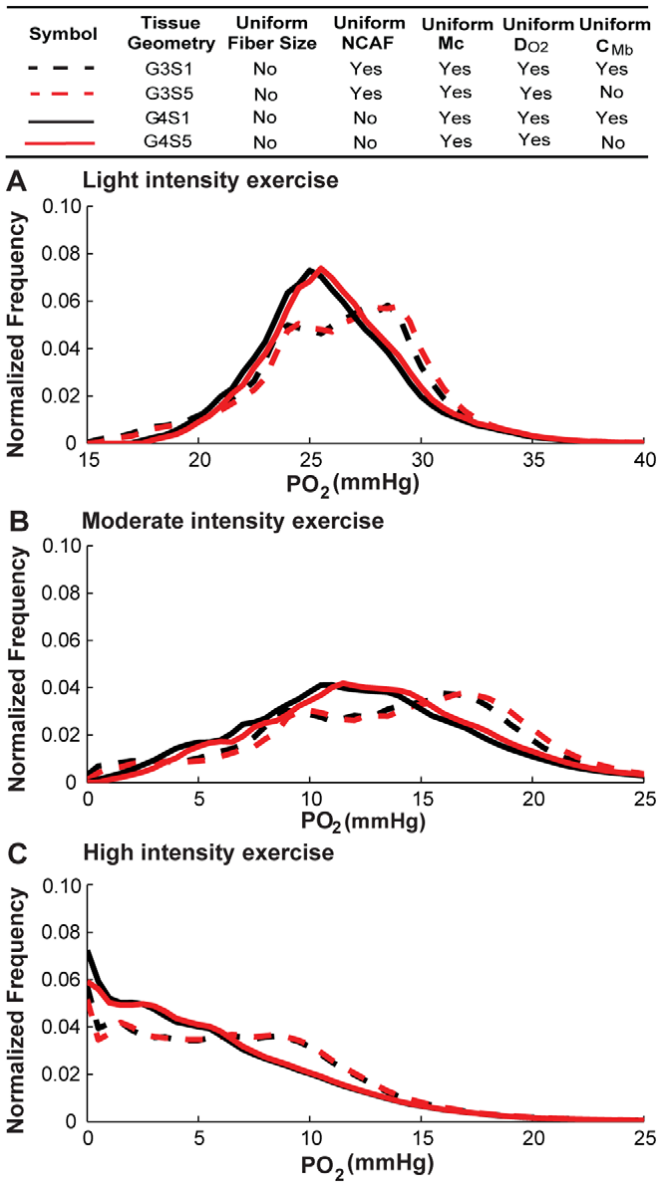
Effects of variation of myoglobin concentration in different fiber types on PO_2_ histograms. Fiber PO_2_ probability distribution profiles in muscle tissues under A) light intensity exercise; B) moderate intensity exercise; and C) high intensity exercise, for simulation cases of G3S1, G3S5, G4S1 and G4S5. Dotted lines are simulation results for G3 geometry with uniform capillary distribution, solid lines are results for G4 geometry with fiber type-dependent distribution. Black lines are simulation scenarios S1 and red lines are simulations scenarios S5 when *C_Mb_* is fiber-type-dependent while others use uniform values for different fiber types.

We next investigated the contribution of the heterogeneity of each fiber-type-dependent variable by itself (*M*_c_, *D_O2_*, and *C_Mb_*) to tissue PO_2_ spatial heterogeneity. To do this, we performed simulations for the non-uniform fiber size geometry with alterations of the factor to be considered (scenarios S3–S5; see Table 3 for a detailed description). Control cases were simulated with uniform fiber properties for comparison. Figures 7, 8, 9 and Table 6 show the simulation results for the effects of *M*_c_, *D_O2_*, *C_Mb_* heterogeneity in different fiber types. The results indicate that the heterogeneity of *M*_c_ in different fiber types (Fig. 7, black vs. red lines) will shift mean PO_2_ levels to lower values (e.g. G3S1 vs. G3S3, 26.5 vs. 25.4 mmHg, light; 13.6 vs. 12.3 mmHg, moderate; 6.9 vs. 5.9 mmHg, high; Table 5), and increase its spatial heterogeneity at all exercise conditions (e.g. G3S1 vs. G3S3, 0.10 vs. 0.16, light; 0.29 vs. 0.48, moderate; 0.65 vs. 0.80, high; Table 5). Fig. 7 shows that the tissue oxygen levels are distributed in a much broader range. Incorporation of heterogeneity of oxygen diffusivity in different fiber types (Fig. 8, black vs. red lines) also affects tissue oxygen profiles. The PO_2_ spatial heterogeneity increased significantly for both G3 (dashed lines) and G4 (solid lines) geometries. However, incorporation of heterogeneity of oxygen diffusivity in different fiber types causes a small increase in the mean PO_2_ level (e.g., G3S1vs G3S4, 26.5 vs. 27.5 mmHg, light; 13.6 vs. 15.04 mmHg, moderate; 6.9 vs. 7.6 mmHg, high; Table 5). Lastly, comparison of simulation results for G3 and G4 geometries with incorporation of myoglobin concentration variation in different fiber types (Fig. 9, black vs. red lines) to control cases indicates that the contribution of C*_Mb_* heterogeneity to PO_2_ spatial heterogeneity is small (Fig. 9 and Table 6). Further investigation of the sensitivity of PO_2_ profile to myoglobin-facilitated diffusion by using a much smaller *D_Mb_* (2×10^−14^ cm^2^/s, i.e. 7 orders of magnitude lower) shows that the tissue PO_2_ profile with no myoglobin-facilitated diffusion does not deviate significantly from control cases at all exercise conditions (Fig. S3).

**Table 6:** Effects of heterogeneities of *M*_c_, *D_O2_*, and *C_Mb_* on oxygen (PO_2_) distribution.

Tissue Geometry*	Exercise**	*P_mean_*	*P_min_*	*P_max_*	*CV(P)*	*%P<1* ***	*%P<2*
G3S3	low	25.36	12.71	43.41	0.16	0.00	0.00
G3S3	medium	12.28	0.03	40.51	0.48	0.03	0.05
G3S3	high	5.87	0.00	38.07	0.80	0.16	0.25
G3S4	low	27.49	17.28	43.88	0.12	0.00	0.00
G3S4	medium	15.04	0.42	41.38	0.37	0.00	0.01
G3S4	high	7.62	0.00	39.20	0.66	0.07	0.13
G3S5	low	26.84	15.70	43.74	0.14	0.00	0.00
G3S5	medium	14.20	0.21	41.12	0.40	0.01	0.02
G3S5	high	7.10	0.00	38.85	0.69	0.09	0.17
G4S3	low	24.82	15.02	43.30	0.15	0.00	0.00
G4S3	medium	10.89	0.13	40.35	0.52	0.01	0.04
G4S3	high	5.25	0.00	38.46	0.93	0.20	0.33
G4S4	low	27.21	19.49	43.44	0.11	0.00	0.00
G4S4	medium	14.31	1.95	40.51	0.33	0.00	0.00
G4S4	high	6.37	0.01	38.17	0.73	0.09	0.17
G4S5	low	26.44	17.93	43.42	0.12	0.00	0.00
G4S5	medium	13.20	0.97	40.50	0.39	0.00	0.00
G4S5	high	6.08	0.01	38.33	0.79	0.12	0.21

* see Table 3 for geometry and scenario annotations.

** Volume-averaged oxygen consumption (*M_c_*) in exercise: low, 3.34×10^−4^ ml O_2_ ml^−1^; medium, 6.68×10^−4^ ml O_2_ ml^−1^; high, 10.02×10^−4^ ml O_2_ ml^−1^.

*** % of tissue that is hypoxic, i.e. with PO_2_<1 or 2 mmHg.

## Discussion and Conclusions

Insufficient oxygen supply from microvascular networks under conditions of increased metabolic demand may lead to new capillary growth from pre-existing microvasculature during physiological conditions (e.g., exercise). Spatial heterogeneity is present in the angiogenic response within exercising muscles. The study of inherent geometric and cellular heterogeneities at the tissue level is critical to a fundamental understanding of organ function and may provide answers for drug resistance and suggestions for efficient therapeutic strategy. The sources of tissue heterogeneity can be attributable to complex microvascular structure and geometry (such as irregular vessel structure, vessel length and diameter distributions), and correspondingly heterogeneous hemodynamic variables such as blood flow rate, shear stress, and hematocrit [40], [41]. These factors have been shown to play an important role in determining heterogeneous oxygen perfusion in tissue using both experimental and theoretical approaches [18], [40], [41]. The effect of the heterogeneity can be difficult to predict without a computational model; for example, inclusion of interstitial space as a separate non-oxygen-consuming volume decreased oxygen heterogeneity from 0.12 to 0.09 in light exercise. However, the heterogeneity in local capillary supply is often correlated with the difference of oxygen consumption capacity in different types of muscle fibers to maintain tissue homeostasis. In this study, we applied a multiscale oxygen transport model to quantitatively evaluate the impact of incorporation of fiber type-specific properties including fiber size on the oxygen distribution. Using rat EDL skeletal muscle as an example, we simulated the distribution of oxygen partial pressure under three exercise conditions (light, moderate, and high intensities) and studied the contributions of fiber type-dependent properties and fiber size to tissue oxygenation profiles.

Our results show that combined effects of fiber type properties other than size do not contribute significantly to tissue oxygen spatial heterogeneity and do not significantly change oxygen profiles compared to the control cases with uniform fiber distribution. By itself, variation of oxygen consumption ratio across the three fiber types has a significant effect (Fig. 7). But that heterogeneity can be compensated by fiber type-specific oxygen diffusivity based on experimentally-measured physiological parameters (Fig. 8); the heterogeneity of myoglobin concentration in different fiber types does not significantly affect PO_2_ profiles (Fig. 9). These three effects are thus collectively small. However, fiber size heterogeneity increases oxygen spatial heterogeneity significantly under all exercise conditions. These data are consistent with experimental observations that oxygen supply is closely correlated with fiber size [42]. In addition, it was shown in rat skeletal muscle that the degree of hypoxia-induced angiogenesis can be due to a fiber size effect [43]. Our results also suggest a small contribution of myoglobin-facilitated diffusion, and consequently differences of myoglobin concentration in different fiber types cause small deviations in oxygen distribution. Its minor contribution at steady state is consistent with other studies [23], [44], [45], although it might play an important role in regulating oxygen PO_2_ at non-equilibrium states and high-intensity exercise conditions [46], [47].

Our model has several limitations. First, current heterogeneous capillary distribution used in the geometry construction is based on the findings that capillary density is primary correlated with fiber oxidative capacity. Other studies have shown that capillary distribution is also a function of fiber size, muscle position (superficial or deep region), muscle type and animal species [48]. These factors can be considered in the future. Second, oxygen distribution in tissue is dependent on the balance of oxygen consumption, oxygen supply and oxygen transport. Thus the parameters related to these variables are critical for tissue PO_2_ profiles. Most of these parameters used in our model are from rat EDL skeletal muscle and thus the conclusions we draw here may only hold true for the EDL. These hypotheses can be tested in other muscle types when muscle-specific parameters are available. Third, in these simulations, we hold blood flow constant (mean capillary velocity at approximately 1 mm/sec, [49]) in order to investigate and dissect the effect of the heterogeneity of fiber type. Fourth, depending on the level of exercise, different muscle fibers are likely recruited. While we do not consider these physiological effects in the current study, the model makes it possible to incorporate such regulatory changes in the future. Fifth, our oxygen transport model is limited to simulation of endurance exercise (primarily aerobic metabolism), but not resistance exercise. Sixth, in our model, exercise is simulated as an increase of oxygen consumption, without taking account the physical contractions and consequent fluid displacement and compartment deformation. Seventh, we do not include remodeling in this study. However, clearly oxygen perfusion is a dynamic process that is regulated by microvascular network geometry, which itself is regulated by oxygen-dependent vascular remodeling. Models that include such a feedback system have been proposed [50], and our current model can be extended to include remodeling. Finally, specific vascular networks may have specific heterogeneous structure that causes blood flow to be imbalanced. Our tissue geometry models are somewhat idealized and actual distribution of hypoxia may be sensitive to the exact placement of vessels – for example, a fiber with six rather than five capillaries nearby may skew the local distribution. However, we hypothesize that significant outliers would be removed by the physiological processes of angiogenesis and vessel regression. We generated two additional sets of tissue geometries and ran all the simulations for these to determine whether the results could be generalized. The conclusions presented here hold also for those additional geometries (see Additional Supplemental Material S2).

In summary, we conclude that differences in fiber size and capillary arrangement (but not other fiber type properties) contribute significantly to oxygen tension profiles in EDL skeletal muscle. Our extended heterogeneous-fiber oxygen transport model can be used to understand local oxygen distribution in other muscle types when fiber-type-specific parameters become available.

## Supporting Information

Supplemental Material S1 **Supporting information text and table S1–2.**
(DOC)

Additional Supplemental Material S2 **Additional supporting information for two additional sets of geometries.**
(DOC)

Figure S1 **Distribution of blood flow rate and hematocrit distribution of the two computed geometries G1 and G2.** Histograms of velocity (A, C) and hematocrit (B,D) distributions for the vascular network G1 and G2. G1 refers to the geometry with uniform fiber size and uniform capillary distribution; G2, uniform fiber size and fiber type-dependent capillary distribution.
(PNG)

Figure S2 **Distribution of blood flow rate and hematocrit distribution of the two computed geometries G3 and G4.** Histograms of velocity (A, C) and hematocrit (B,D) distributions for the vascular networks G3 and G4. G3 refers to the geometry with non-uniform fiber size and uniform capillary distribution; G4, non-uniform fiber size and fiber type-dependent capillary distribution.
(PNG)

Figure S3 **Effects of fiber-type specific myoglobin diffusivity on oxygen distribution in geometries of non-uniform-size fibers.** Fiber PO2 under A) light intensity exercise, volume-averaged *m_c_* = 3.64×10^−4^ mlO2 ml^−1^ s^−1^; B) moderate intensity exercise, volume-averaged *m_c_* = 6.68×10^−4^ mlO2 ml^−1^ s^−1^; and C) high intensity exercise, volume-averaged *m_c_* = 1.02×10^−3^ mlO2 ml^−1^ s^−1^ in muscle tissue of G3S1,G3S6,G4S1,G3S6. Dashed lines are simulations for G3 geometry with uniform capillary considered; solid lines are results from G4 geometry with fiber type-dependent capillary distribution. Black lines are the simulation cases (S1) with uniform fiber-type properties (*m_c_*, *D_O2_*,*C_Mb_*) and red lines are simulations when *D_Mb_* uses low value 3×10^−14^ instead of 3×10^−7^ (S6).
(PNG)
