# Supplementary material for: Effects of Fiber Type and Size on the Heterogeneity of Oxygen Distribution in Exercising Skeletal Muscle
Source: PLoS One. 2012 Sep 18;7(9):e44375. doi: 10.1371/journal.pone.0044375 (PMC3445540; doi:10.1371/journal.pone.0044375)
Supplement: Supplemental Material S1 — Supporting information text and table S1–2. (DOC) [file pone.0044375.s001.doc]

Supplement to:

# Effects of fiber type and size on the heterogeneity

# of oxygen distribution in exercising skeletal muscle

Gang Liu, Feilim Mac Gabhann, Aleksander S. Popel

**Contents**

***Supplemental Tables***

Table S1. Number of capillaries surrounding a fiber (NCAF) for each fiber type in the four computed geometries.

Table S2. Flow characteristics of the four computed geometries.

**Table S1. Number of capillaries surrounding a fiber (NCAF)* for each fiber type in the four computed tissue geometries**

| **Geometry**** | **Type I** | **Type IIa** | **Type IIb** |
| --- | --- | --- | --- |
| **G1** | 3.25+0.15 | 3.27 ± 0.14 | 3.24 ± 0.14 |
| **G2** | 5.25 ± 0.27 | 4.18 ± 0.19 | 2.67 ± 0.33 |
| **G3** | 3.5 ± 0.16 | 3.15 ± 0.25 | 3.20 ± 0.25 |
| **G4** | 4.75 ± 0.20 | 3.63 ± 0.30 | 3.09 ± 0.44 |

*Note that unlike the *capillary-to-fiber ratio* metric (C:F), the NCAF values do allow double-counting of capillaries; i.e. the whole number of capillaries neighboring a fiber is counted, regardless of whether those capillaries are shared with other fibers. The cross section when z=400 µm is used to compute NCAF.

**Tissue geometry: G1, uniform fiber size and uniform capillary distribution; G2, uniform fiber size and fiber type-dependent capillary distribution; G3, non-uniform fiber size and uniform capillary distribution; G4, uniform fiber size and uniform capillary distribution. Values are reported as mean (across all fibers of that type in a control tissue), ± standard deviation.

**Table S2. Flow characteristics: Total blood Flow rate (*Q_in_*, ml blood per 100 g tissue per min), mean blood flow velocity (*v_a_*, µm per s), and mean discharge hematocrit (*H_da_*) across the vascular network in the four computed geometries.**

| ***Geometry**** | ***Q_in_*** | ***v_a_*** | ***CV(v)*** | ***H_da_*** | ***CV(H_d_)*** |
| --- | --- | --- | --- | --- | --- |
| G1 | 165.7 | 1269 | 0.128 | 0.41 | 0.25 |
| G2 | 164.5 | 1269 | 0.129 | 0.41 | 0.25 |
| G3 | 167.1 | 1266 | 0.127 | 0.41 | 0.26 |
| G4 | 167.9 | 1270 | 0.123 | 0.41 | 0.26 |
|  |  |  |  |  |  |

*Tissue geometry: G1, uniform fiber size and uniform capillary distribution; G2, uniform fiber size and fiber type-dependent capillary distribution; G3, non-uniform fiber size and uniform capillary distribution; G4, uniform fiber size and uniform capillary distribution.
