# Supplementary material for: Effects of Fiber Type and Size on the Heterogeneity of Oxygen Distribution in Exercising Skeletal Muscle
Source: PLoS One. 2012 Sep 18;7(9):e44375. doi: 10.1371/journal.pone.0044375 (PMC3445540; doi:10.1371/journal.pone.0044375)
Supplement: Additional Supplemental Material S2 — Additional supporting information for two additional sets of geometries. (DOC) [file pone.0044375.s002.doc]

**Supplement to:**

# Effects of fiber type and size on the heterogeneity

# of oxygen distribution in exercising skeletal muscle

Gang Liu, Feilim Mac Gabhann, Aleksander S. Popel

**Generalization of results: alternate tissue geometries**

To test whether or not the conclusions can be generalized beyond the specific configurations as shown in the manuscript, we have generated two additional sets of geometries using the algorithm defined in the manuscript (Figures SA1 and SA2). The results derived from them are collated here (Figures SA3-SA7, Table SA1-SA5). Each of the two sets of tissue structures has four geometries analogous to the G1-G4 geometries in the manuscript (recall: G1 = uniform fiber size, uniform capillary distribution; G2 = uniform fiber size, non-uniform capillary distribution; G3 = nonuniform fiber size, uniform capillary distribution; G4 = non-uniform fiber size, non-uniform capillary distribution). 2D cross sections of the geometries are shown in Fig. SA1 and SA2. The corresponding number of capillaries around fibers (NCAF) and flow rates are shown in Table SA1 and SA2.

We ran a complete set of simulations (recall: denoted S1, S2, S3, S4, S5 in the main manuscript) for each of the geometries in these two sets (i.e. for G1, G2, G3 and G4 in Sample Sets 2 and Sample Sets 3). Comparing these results to the figures in main manuscripts reinforces the conclusions drawn from the original figures based on the first set of geometries. These conclusions are: (1) the combined effects of fiber type properties other than size do not contribute significantly to tissue oxygen spatial heterogeneity and do not significantly change oxygen profiles compared to the control cases with uniform fiber distribution; (2) heterogeneity of fiber size does contribute significantly to the heterogeneity of oxygen distribution; (3) heterogeneity of capillary distribution contributes significantly to the heterogeneity of oxygen distribution.

In sum, we conclude our results can be generalized beyond the specific configuration from the current manuscript.

Specific conclusions for each of the figures included in the additional supplemental file are as follows:

*i) Figure SA3 (Compare to Figure 3 in main manuscript):*

When fiber size is uniformly distributed, fiber type properties other than size do not contribute significantly to tissue oxygen spatial heterogeneity (compare S1 to S2, i.e. red to black). However, capillary distribution *does* contribute tissue oxygen spatial heterogeneity (compare G1 to G2, i.e. solid to dashed lines). Specifically, capillaries arranged in a fiber-type-specific manner reduce the average oxygen level, while causing a slight (5-12%) increase in the coefficient of variation (CV).

*ii) Figure SA4 (Compare to Figure 4 in main manuscript):*

Similar to Figure SA2, when fiber size in not uniformly distributed, fiber type properties other than size also do not contribute significantly to tissue oxygen spatial heterogeneity. Again, capillary distribution *does* contribute tissue oxygen spatial heterogeneity (compare G3 to G4, i.e. solid to dashed lines). Specifically, capillaries arranged in a fiber-type-specific manner reduce the average oxygen level, while causing a slight increase in the coefficient of variation.

*iii) Figures SA3 and SA4 (Compare to Figures 3 and 4 in main manuscript):*

The effect of fiber size heterogeneity can be observed by comparing the results of G3 to G1 and G4 to G2. Fiber size heterogeneity decreases the average oxygen level by approximately 8-25% while increasing coefficient of variation by 30-50%.

*iv) Figure SA5 (Compare to Figure 7 in main manuscript):*

When fiber size in not uniformly distributed, heterogeneity of fiber-specific oxygen consumption rate alone does contribute significantly to tissue oxygen spatial heterogeneity.

*v) Figure SA6 (Compare to Figure 8 in main manuscript):*

When fiber size in not uniformly distributed, heterogeneity of oxygen diffusion rate in different fiber types does contribute significantly to tissue oxygen spatial heterogeneity. Note that this contribution tends to balance out that of fiber-specific oxygen consumption rate.

*vi) Figure SA7 (Compare to Figure 9 in main manuscript):*

When fiber size in not uniformly distributed, heterogeneity of myoglobin concentration in different fiber types does not contribute significantly to tissue oxygen spatial heterogeneity.

**List of results for Sample Sets 2 and 3:**

**Figure SA1.** Sample Geometries G1-4 for Sample Set 2.

**Figure SA2.** Sample Geometries G1-4 for Sample Set 3.

**Figure SA3.** Uniform fiber size: effect of fiber-type specific number of capillaries around a fiber (NCAF) (G2) vs. uniform capillary density (G1).

**Figure SA4.** Type-specific fiber size: effect of fiber-type specific number of capillaries around a fiber (NCAF) (G4) vs. uniform capillary density (G3)

**Figure SA5.** Heterogeneous fiber size: effect of fiber-type specific oxygen consumption rate (Mc) (S3) vs. uniform consumption (S1).

**Figure SA6.** Heterogeneous fiber size: effect of fiber-type specific oxygen diffusivity (D_O2_) (S4) vs. uniform diffusivity (S1).

**Figure SA7.** Heterogeneous fiber size: effect of fiber-type specific myoglobin concentration (C_Mb_) (S5) vs. uniform concentration (S1).

**Table SA1.** Number of capillaries surrounding a fiber (NCAF) for each fiber type in the four computed geometries: values for Sample Sets 2 and 3.

**Table SA2.** Flow characteristics of the four computed geometries: Total blood Flow rate (*Q_in_*), mean blood flow velocity (*v_a_*), and mean hematocrit (*H_da_*) across the vascular network.

**Table SA3.** Effects of fiber type properties on oxygen (PO_2_) distribution in sample set 2 (Uniform fiber size) .

**Table SA4.** Effects of fiber type properties on oxygen (PO_2_) distribution in sample set 3 (Uniform fiber size).

**Table SA5.** Effects of fiber type properties on oxygen (PO_2_) distribution in sample set 2 (Fiber type specific fiber size).

**Table SA6.** Effects of fiber type properties on oxygen (PO_2_) distribution in sample set 3 (Fiber type specific fiber size).

**Table SA7.** Effects of heterogeneities of *M*_c_, *D_O2_,* and *C_Mb_* on oxygen (PO_2_) distribution in sample set 2.

**Table SA8.** Effects of heterogeneities of *M*_c_, *D_O2_,* and *C_Mb_* on oxygen (PO_2_) distribution in sample set 3.

**Figure SA1. Sample Geometries G1-4 for Sample Set 2.**

[Compare to Geometries for Sample Set 1 in Manuscript Figures 1 and 2]


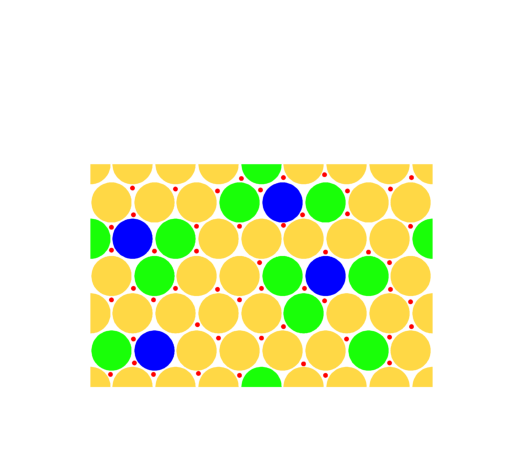

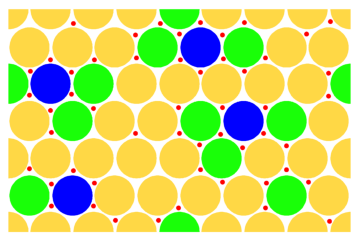
**G1 G2**


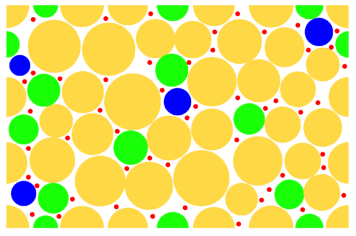

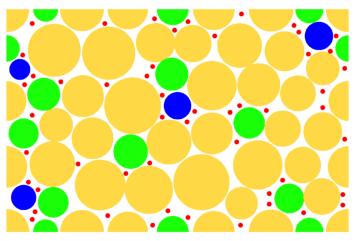
**G3 G4**

**Figure SA2. Sample Geometries G1-4 for Sample Set 3.**

[Compare to Geometries for Sample Set 1 in Manuscript Figures 1 and 2]


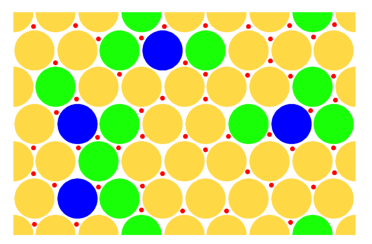

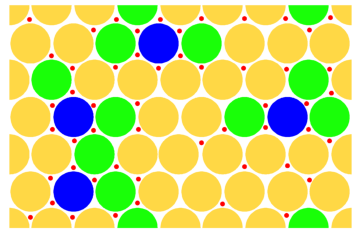
**G1 G2**


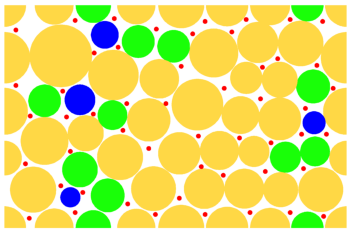

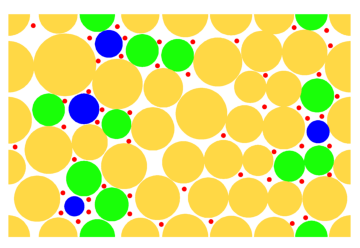
**G3 G4**

**Figure SA3. Uniform fiber size: effect of fiber-type specific number of capillaries around a fiber (NCAF) (G2) vs. uniform capillary density (G1).**

[Compare to results for Sample Set 1 in Manuscript Figure 3]


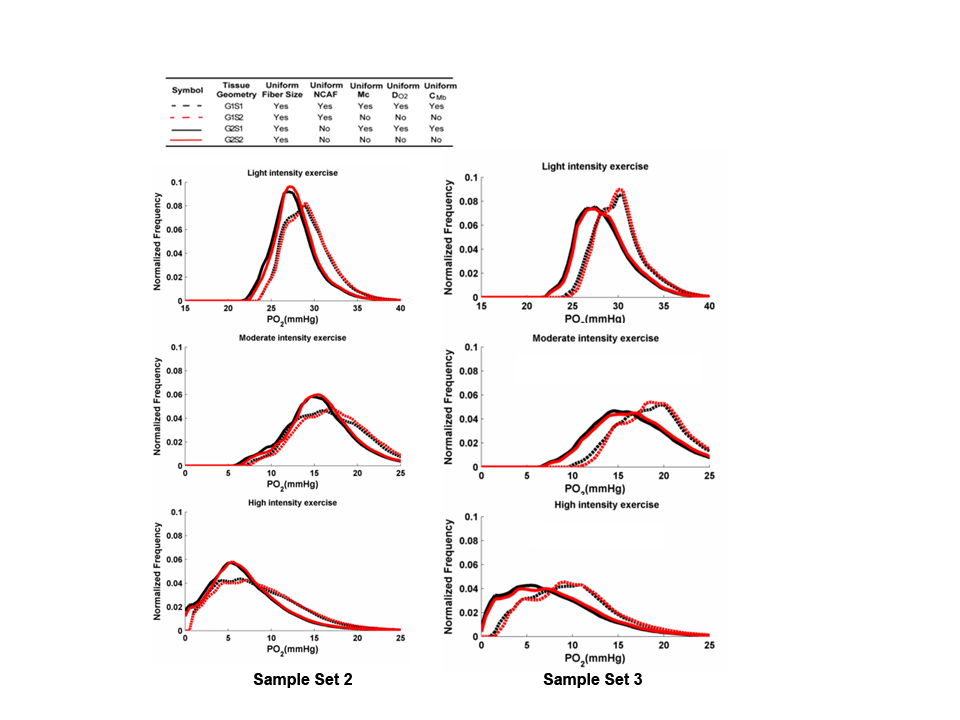


**Sample Set 2 Sample Set 3**

**Figure SA4. Heterogeneous fiber size: effect of fiber-type specific number of capillaries around a fiber (NCAF) (G4) vs. uniform capillary density (G3)**

[Compare to results for Sample Set 1 in Manuscript Figure 4]

**
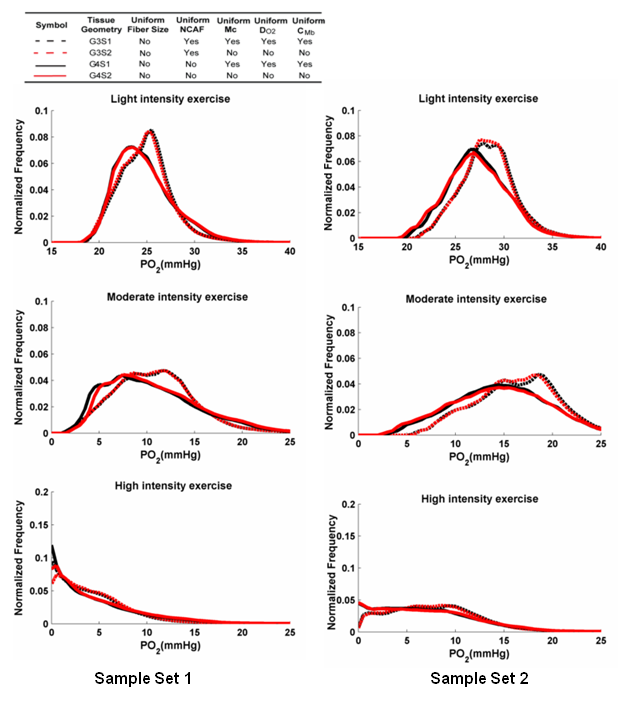
**

**Sample Set 2 Sample Set 3**

**Figure SA5. Heterogeneous fiber size: effect of fiber-type specific oxygen consumption rate (Mc) (S3) vs. uniform consumption (S1).**

[Compare to results for Sample Set 1 in Manuscript Figure 7]


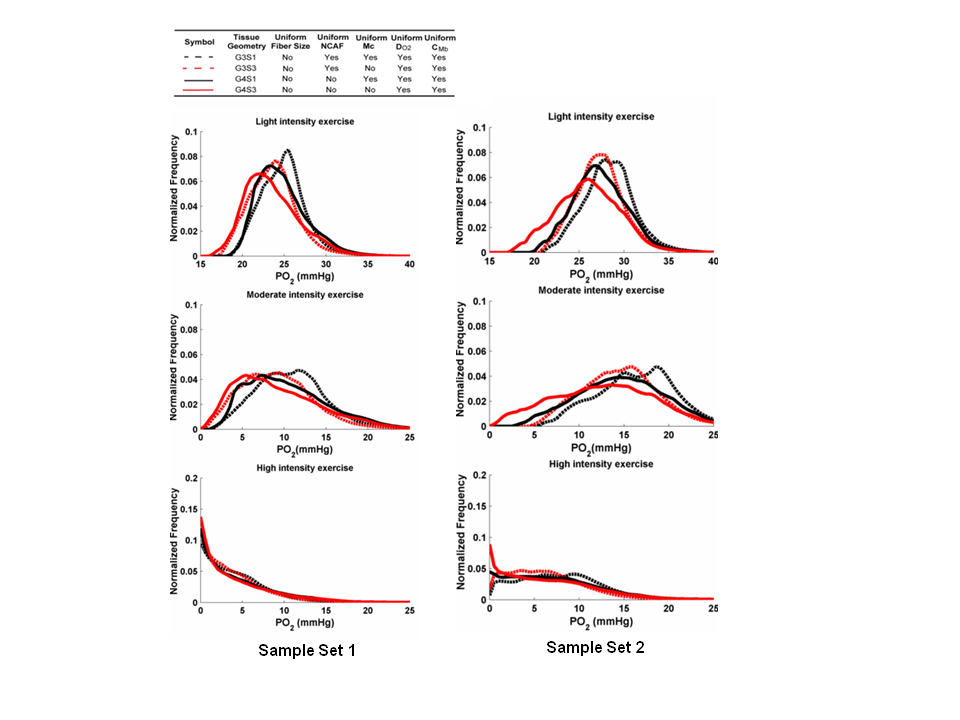


**Sample Set 2 Sample Set 3**

**Figure SA6. Heterogeneous fiber size: effect of fiber-type specific oxygen diffusivity (D_O2_) (S4) vs. uniform diffusivity (S1).**

[Compare to results for Sample Set 1 in Manuscript Figure 8]

**
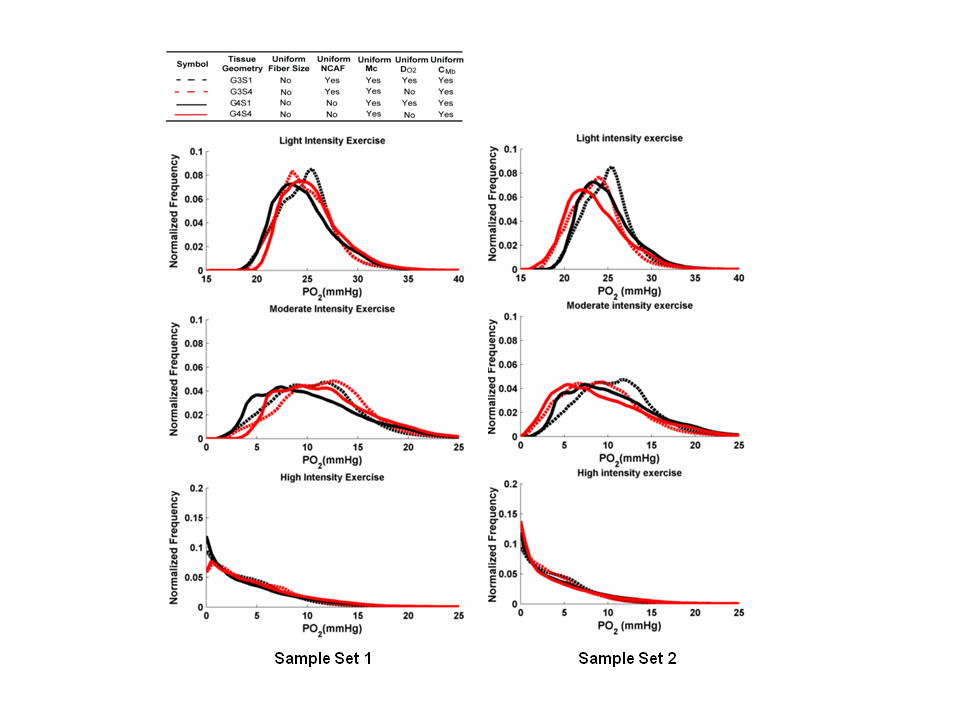
**

**Sample Set 2 Sample Set 3**

**Figure SA7. Heterogeneous fiber size: effect of fiber-type specific myoglobin concentration (C_Mb_) (S5) vs. uniform concentration (S1).**

[Compare to results for Sample Set 1 in Manuscript Figure 9]

**
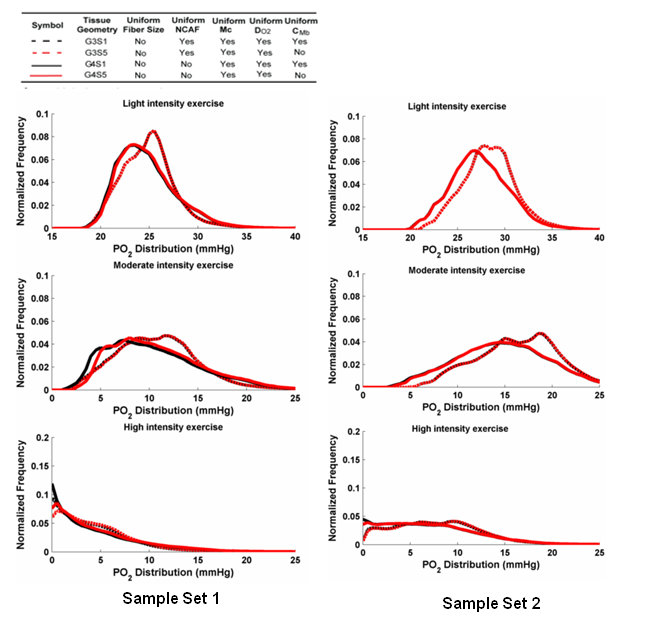
**

**Sample Set 2 Sample Set 3**

**Table SA1. Number of capillaries surrounding a fiber (NCAF) for each fiber type in the four computed geometries: values for Sample Sets 2 and 3.**

[Compare to values for Sample Set 1 in Supplemental Material Table S1]

| **Sample Set** | **Geometry** | **Type I** | **Type IIa** | **Type IIb** |
| --- | --- | --- | --- | --- |
| 2 | G1 | 3.5+0.16 | 3.36+0.15 | 3.09+0.20 |
| 2 | G2 | 5.5+0.10 | 4.27+0.18 | 2.54+0.36 |
| 2 | G3 | 3+0.27 | 2.9+0.39 | 3.24+0.27 |
| 2 | G4 | 4.5+0.20 | 4+0.27 | 3.06+0.42 |
| 3 | G1 | 3.25+0.15 | 3.27+0.20 | 3.21+0.25 |
| 3 | G2 | 5.25+0.18 | 4.27+0.15 | 2.66+0.34 |
| 3 | G3 | 3+0.0 | 2.9+0.18 | 3.3+0.26 |
| 3 | G4 | 4.75+0.10 | 4+0.19 | 2.8+0.33 |

**Table SA2. Flow characteristics of the four computed geometries: Total blood Flow rate (*Q_in_,* ml blood per 100 g tissue per min), mean blood flow velocity (*v_a_,* µm per s), and mean hematocrit (*H_da_*) across the vascular network.**

[Compare to values for Sample Set 1 in Supplemental Material Table S2]

| **Sample Set** | **Geometry** | **Qin** | **va** | **CV(v)** | **Hda** | **CV(Hd)** |
| --- | --- | --- | --- | --- | --- | --- |
| 2 | G1 | 156.5 | 1229 | 0.12 | 0.412 | 0.25 |
| 2 | G2 | 158.4 | 1234 | 0.11 | 0.409 | 0.24 |
| 2 | G3 | 157.2 | 1244 | 0.13 | 0.41 | 0.28 |
| 2 | G4 | 161 | 1257 | 0.14 | 0.41 | 0.28 |
| 3 | G1 | 170.25 | 1240.3 | 0.15 | 0.41 | 0.29 |
| 3 | G2 | 168.4 | 1235.7 | 0.14 | 0.41 | 0.28 |
| 3 | G3 | 177 | 1261.05 | 0.14 | 0.41 | 0.28 |
| 3 | G4 | 175 | 1260 | 0.14 | 0.41 | 0.27 |

**Table SA3. Effects of fiber type properties on oxygen (PO_2_) distribution in sample set two (Uniform Fiber Size).**

[Compare to results for Sample Set 1 in manuscript Table 4]

| **Tissue Geometry*** | **Exercise**** | ***P_mean_*** | ***P_min_*** | ***P****_max_* | ***CV(P)*** | ***%P<1****** | ***%P<2*** |
| --- | --- | --- | --- | --- | --- | --- | --- |
| G1S1 | low | 28.40 | 22.95 | 43.04 | 0.09 | 0.00 | 0.00 |
| G1S1 | medium | 16.23 | 6.40 | 39.77 | 0.27 | 0.00 | 0.00 |
| G1S1 | high | 7.71 | 0.09 | 36.99 | 0.63 | 0.04 | 0.09 |
| G1S2 | low | 28.55 | 22.98 | 43.06 | 0.09 | 0.00 | 0.00 |
| G1S2 | medium | 16.79 | 6.87 | 39.85 | 0.26 | 0.00 | 0.00 |
| G1S2 | high | 8.06 | 0.11 | 37.05 | 0.61 | 0.03 | 0.08 |
| G2S1 | low | 27.92 | 22.26 | 42.26 | 0.09 | 0.00 | 0.00 |
| G2S1 | medium | 15.84 | 6.10 | 39.09 | 0.25 | 0.00 | 0.00 |
| G2S1 | high | 7.10 | 0.08 | 36.31 | 0.60 | 0.04 | 0.09 |
| G2S2 | low | 28.21 | 22.70 | 42.30 | 0.09 | 0.00 | 0.00 |
| G2S2 | medium | 16.22 | 6.61 | 39.13 | 0.24 | 0.00 | 0.00 |
| G2S2 | high | 7.36 | 0.12 | 36.32 | 0.58 | 0.03 | 0.07 |

* see Manuscript Table 3 for geometry and scenario annotations

**Volume-averaged oxygen consumption (*M*_c_) in exercise: low, 3.34 x10^-4^ ml O_2_ ml^-1^; medium, 6.68 x10^-4^ ml O_2_ ml^-1^; high, 10.02 x10^-4^ ml O_2_ ml^-1^

*** % of tissue that is hypoxic, i.e. with PO_2_ < 1 or 2 mmHg

**Table SA4. Effects of fiber type properties on oxygen (PO_2_) distribution in sample set three (Uniform Fiber Size).**

[Compare to results for Sample Set 1 in manuscript Table 4]

| **Tissue Geometry*** | **Exercise**** | ***P_mean_*** | ***P_min_*** | ***P****_max_* | ***CV(P)*** | ***%P<1****** | ***%P<2*** |
| --- | --- | --- | --- | --- | --- | --- | --- |
| G1S1 | low | 29.33 | 23.38 | 43.03 | 0.09 | 0.00 | 0.00 |
| G1S1 | medium | 18.31 | 9.01 | 40.42 | 0.22 | 0.00 | 0.00 |
| G1S1 | high | 9.33 | 0.55 | 38.00 | 0.52 | 0.00 | 0.03 |
| G1S2 | low | 29.57 | 23.91 | 43.05 | 0.09 | 0.00 | 0.00 |
| G1S2 | medium | 18.74 | 10.00 | 40.45 | 0.21 | 0.00 | 0.00 |
| G1S2 | high | 9.65 | 0.83 | 38.02 | 0.50 | 0.00 | 0.02 |
| G2S1 | low | 28.60 | 22.36 | 42.65 | 0.10 | 0.00 | 0.00 |
| G2S1 | medium | 16.87 | 6.84 | 39.45 | 0.27 | 0.00 | 0.00 |
| G2S1 | high | 7.98 | 0.19 | 36.90 | 0.63 | 0.03 | 0.09 |
| G2S2 | low | 28.82 | 22.44 | 42.81 | 0.10 | 0.00 | 0.00 |
| G2S2 | medium | 17.28 | 7.05 | 39.63 | 0.26 | 0.00 | 0.00 |
| G2S2 | high | 8.40 | 0.24 | 37.15 | 0.61 | 0.02 | 0.08 |

* see Manuscript Table 3 for geometry and scenario annotations

**Volume-averaged oxygen consumption (*M*_c_) in exercise: low, 3.34 x10^-4^ ml O_2_ ml^-1^; medium, 6.68 x10^-4^ ml O_2_ ml^-1^; high, 10.02 x10^-4^ ml O_2_ ml^-1^

*** % of tissue that is hypoxic, i.e. with PO_2_ < 1 or 2 mmHg

**Table SA5. Effects of fiber type properties on oxygen (PO_2_) distribution in sample set two (Fiber type specific fiber size)**

[Compare to results for Sample Set 1 in manuscript Table 5]

| **Tissue Geometry*** | **Exercise**** | ***P_mean_*** | ***P_min_*** | ***P****_max_* | ***CV(P)*** | ***%P<1****** | ***%P<2*** |
| --- | --- | --- | --- | --- | --- | --- | --- |
| G3S1 | low | 25.13 | 19.02 | 40.82 | 0.11 | 0.00 | 0.00 |
| G3S1 | medium | 11.11 | 1.69 | 36.54 | 0.39 | 0.00 | 0.00 |
| G3S1 | high | 4.52 | 0.01 | 34.14 | 0.86 | 0.18 | 0.31 |
| G3S2 | low | 24.99 | 18.87 | 40.70 | 0.11 | 0.00 | 0.00 |
| G3S2 | medium | 11.13 | 1.86 | 36.77 | 0.38 | 0.00 | 0.00 |
| G3S2 | high | 4.79 | 0.03 | 34.44 | 0.81 | 0.13 | 0.27 |
| G4S1 | low | 24.98 | 18.70 | 41.41 | 0.12 | 0.00 | 0.00 |
| G4S1 | medium | 10.73 | 1.54 | 37.71 | 0.46 | 0.00 | 0.00 |
| G4S1 | high | 4.79 | 0.02 | 35.92 | 0.94 | 0.21 | 0.35 |
| G4S2 | low | 25.03 | 18.49 | 41.46 | 0.12 | 0.00 | 0.00 |
| G4S2 | medium | 11.17 | 1.93 | 37.78 | 0.44 | 0.00 | 0.00 |
| G4S2 | high | 5.08 | 0.04 | 35.95 | 0.89 | 0.17 | 0.31 |

* * see Manuscript Table 3 for geometry and scenario annotations

**Volume-averaged oxygen consumption (*M*_c_) in exercise: low, 3.34 x10^-4^ ml O_2_ ml^-1^; medium, 6.68 x10^-4^ ml O_2_ ml^-1^; high, 10.02 x10^-4^ ml O_2_ ml^-1^

*** % of tissue that is hypoxic, i.e. with PO_2_ < 1 or 2 mmHg

**Table SA6. Effects of fiber type properties on oxygen (PO_2_) distribution in sample set three (Fiber type specific fiber size)**

[Compare to results for Sample Set 1 in manuscript Table 5]

| **Tissue Geometry*** | **Exercise**** | ***P_mean_*** | ***P_min_*** | ***P****_max_* | ***CV(P)*** | ***%P<1****** | ***%P<2*** |
| --- | --- | --- | --- | --- | --- | --- | --- |
| G3S1 | Low | 28.57 | 21.06 | 42.02 | 0.11 | 0.00 | 0.00 |
| G3S1 | medium | 16.76 | 5.40 | 38.24 | 0.27 | 0.00 | 0.00 |
| G3S1 | high | 8.13 | 0.15 | 34.93 | 0.58 | 0.03 | 0.09 |
| G3S2 | low | 28.50 | 21.41 | 41.95 | 0.11 | 0.00 | 0.00 |
| G3S2 | medium | 16.65 | 5.91 | 38.12 | 0.26 | 0.00 | 0.00 |
| G3S2 | high | 8.04 | 0.19 | 34.85 | 0.57 | 0.03 | 0.09 |
| G4S1 | low | 27.59 | 20.25 | 43.70 | 0.11 | 0.00 | 0.00 |
| G4S1 | medium | 15.09 | 3.19 | 41.49 | 0.33 | 0.00 | 0.00 |
| G4S1 | high | 7.31 | 0.03 | 39.84 | 0.69 | 0.09 | 0.16 |
| G4S2 | low | 27.43 | 19.72 | 43.73 | 0.12 | 0.00 | 0.00 |
| G4S2 | medium | 14.90 | 2.68 | 41.56 | 0.35 | 0.00 | 0.00 |
| G4S2 | high | 7.42 | 0.03 | 39.97 | 0.70 | 0.09 | 0.16 |

* see Manuscript Table 3 for geometry and scenario annotations

**Volume-averaged oxygen consumption (*M*_c_) in exercise: low, 3.34 x10^-4^ ml O_2_ ml^-1^; medium, 6.68 x10^-4^ ml O_2_ ml^-1^; high, 10.02 x10^-4^ ml O_2_ ml^-1^

*** % of tissue that is hypoxic, i.e. with PO_2_ < 1 or 2 mmHg

**Table SA7. Effects of heterogeneities of *M*_c_, *D_O2_,* and *C_Mb_* on oxygen (PO_2_) distribution in sample set two**

[Compare to results for Sample Set 1 in manuscript Table 6]

| **Tissue Geometry*** | **Exercise** | ***P_mean_*** | ***P_min_*** | ***P****_max_* | ***CV(P)*** | ***%P<1****** | ***%P<2*** |
| --- | --- | --- | --- | --- | --- | --- | --- |
| G3S3 | low | 23.95 | 17.48 | 40.55 | 0.12 | 0.00 | 0.00 |
| G3S3 | medium | 9.47 | 0.75 | 36.68 | 0.47 | 0.00 | 0.01 |
| G3S3 | high | 4.18 | 0.01 | 34.59 | 0.90 | 0.20 | 0.34 |
| G3S4 | low | 25.05 | 19.49 | 40.77 | 0.11 | 0.00 | 0.00 |
| G3S4 | medium | 12.06 | 2.28 | 36.71 | 0.35 | 0.00 | 0.00 |
| G3S4 | high | 5.05 | 0.04 | 34.09 | 0.83 | 0.13 | 0.27 |
| G3S5 | low | 25.14 | 19.03 | 40.83 | 0.11 | 0.00 | 0.00 |
| G3S5 | medium | 11.20 | 1.87 | 36.56 | 0.38 | 0.00 | 0.00 |
| G3S5 | high | 4.83 | 0.04 | 34.16 | 0.80 | 0.13 | 0.27 |
| G4S3 | low | 23.83 | 16.48 | 41.45 | 0.14 | 0.00 | 0.00 |
| G4S3 | medium | 9.54 | 0.55 | 37.66 | 0.54 | 0.00 | 0.02 |
| G4S3 | high | 4.54 | 0.01 | 35.80 | 0.98 | 0.24 | 0.38 |
| G4S4 | low | 25.68 | 20.13 | 41.38 | 0.11 | 0.00 | 0.00 |
| G4S4 | medium | 12.13 | 3.47 | 37.99 | 0.38 | 0.00 | 0.00 |
| G4S4 | high | 5.35 | 0.08 | 35.92 | 0.84 | 0.14 | 0.27 |
| G4S5 | low | 25.09 | 18.87 | 41.43 | 0.12 | 0.00 | 0.00 |
| G4S5 | medium | 11.26 | 2.40 | 37.80 | 0.43 | 0.00 | 0.00 |
| G4S5 | high | 5.07 | 0.06 | 35.92 | 0.88 | 0.16 | 0.30 |

* see Manuscript Table 3 for geometry and scenario annotations

**Volume-averaged oxygen consumption (*M*_c_) in exercise: low, 3.34 x10^-4^ ml O_2_ ml^-1^; medium, 6.68 x10^-4^ ml O_2_ ml^-1^; high, 10.02 x10^-4^ ml O_2_ ml^-1^

*** % of tissue that is hypoxic, i.e. with PO_2_ < 1 or 2 mmHg

**Table SA8. Effects of heterogeneities of *M*_c_, *D_O2_,* and *C_Mb_* on oxygen (PO_2_) distribution in sample set three**

[Compare to results for Sample Set 1 in manuscript Table 6]

| **Tissue Geometry*** | **Exercise**** | ***P_mean_*** | ***P_min_*** | ***P****_max_* | ***CV(P)*** | ***%P<1****** | ***%P<2*** |
| --- | --- | --- | --- | --- | --- | --- | --- |
| G3S3 | low | 27.59 | 21.01 | 41.57 | 0.10 | 0.00 | 0.00 |
| G3S3 | medium | 14.96 | 4.90 | 37.39 | 0.29 | 0.00 | 0.00 |
| G3S3 | high | 6.59 | 0.11 | 34.05 | 0.66 | 0.05 | 0.14 |
| G3S4 | low | 28.96 | 20.19 | 42.26 | 0.10 | 0.00 | 0.00 |
| G3S4 | medium | 17.59 | 4.43 | 38.73 | 0.28 | 0.00 | 0.00 |
| G3S4 | high | 9.27 | 0.13 | 35.62 | 0.54 | 0.03 | 0.08 |
| G3S5 | low | 28.59 | 21.05 | 42.02 | 0.10 | 0.00 | 0.00 |
| G3S5 | medium | 16.81 | 5.27 | 38.26 | 0.27 | 0.00 | 0.00 |
| G3S5 | high | 8.28 | 0.12 | 34.96 | 0.57 | 0.03 | 0.09 |
| G4S3 | low | 26.27 | 17.57 | 43.67 | 0.13 | 0.00 | 0.00 |
| G4S3 | medium | 13.06 | 0.69 | 41.48 | 0.44 | 0.00 | 0.01 |
| G4S3 | high | 6.61 | 0.01 | 40.03 | 0.79 | 0.14 | 0.23 |
| G4S4 | low | 28.25 | 21.77 | 43.72 | 0.10 | 0.00 | 0.00 |
| G4S4 | medium | 16.24 | 5.48 | 41.35 | 0.28 | 0.00 | 0.00 |
| G4S4 | high | 7.78 | 0.06 | 39.57 | 0.64 | 0.05 | 0.11 |
| G4S5 | low | 27.61 | 20.29 | 43.70 | 0.11 | 0.00 | 0.00 |
| G4S5 | medium | 15.19 | 3.47 | 41.50 | 0.33 | 0.00 | 0.00 |
| G4S5 | high | 7.42 | 0.04 | 39.85 | 0.68 | 0.07 | 0.15 |

* see Manuscript Table 3 for geometry and scenario annotations

**Volume-averaged oxygen consumption (*M*_c_) in exercise: low, 3.34 x10^-4^ ml O_2_ ml^-1^; medium, 6.68 x10^-4^ ml O_2_ ml^-1^; high, 10.02 x10^-4^ ml O_2_ ml^-1^

*** % of tissue that is hypoxic, i.e. with PO_2_ < 1 or 2 mmHg
