# Supplementary figures and images for: Effects of Fiber Type and Size on the Heterogeneity of Oxygen Distribution in Exercising Skeletal Muscle
Source: PLoS One. 2012 Sep 18;7(9):e44375. doi: 10.1371/journal.pone.0044375 (PMC3445540; doi:10.1371/journal.pone.0044375)

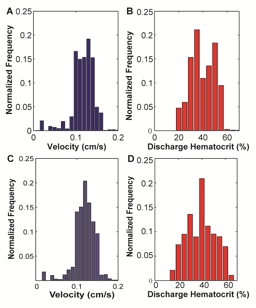

Supplement: Figure S1 — Distribution of blood flow rate and hematocrit distribution of the two computed geometries G1 and G2. Histograms of velocity (A, C) and hematocrit (B,D) distributions for the vascular network G1 and G2. G1 refers to the geometry with uniform fiber size and uniform capillary distribution; G2, uniform fiber size and fiber type-dependent capillary distribution. (PNG) [file pone.0044375.s003.png]

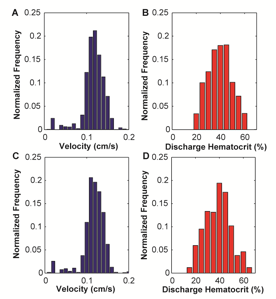

Supplement: Figure S2 — Distribution of blood flow rate and hematocrit distribution of the two computed geometries G3 and G4. Histograms of velocity (A, C) and hematocrit (B,D) distributions for the vascular networks G3 and G4. G3 refers to the geometry with non-uniform fiber size and uniform capillary distribution; G4, non-uniform fiber size and fiber type-dependent capillary distribution. (PNG) [file pone.0044375.s004.png]

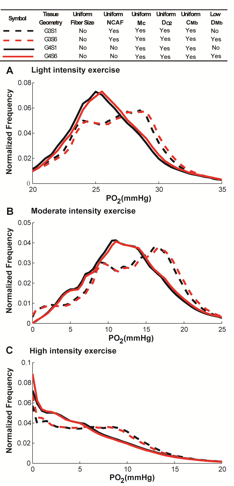

Supplement: Figure S3 — Effects of fiber-type specific myoglobin diffusivity on oxygen distribution in geometries of non-uniform-size fibers. Fiber PO2 under A) light intensity exercise, volume-averaged mc = 3.64×10^−4^ mlO2 ml−1 s−1; B) moderate intensity exercise, volume-averaged mc = 6.68×10^−4^ mlO2 ml−1 s−1; and C) high intensity exercise, volume-averaged mc = 1.02×10^−3^ mlO2 ml−1 s−1 in muscle tissue of G3S1,G3S6,G4S1,G3S6. Dashed lines are simulations for G3 geometry with uniform capillary considered; solid lines are results from G4 geometry with fiber type-dependent capillary distribution. Black lines are the simulation cases (S1) with uniform fiber-type properties (mc, DO2,CMb) and red lines are simulations when DMb uses low value 3×10^−14^ instead of 3×10^−7^ (S6). (PNG) [file pone.0044375.s005.png]
